# The effect of inosine on the spectroscopic properties and crystal structure of a NIR-emitting DNA-stabilized silver nanocluster†

**DOI:** 10.1039/d2na00325b

**Published:** 2022-06-20

**Authors:** Cecilia Cerretani, Mikkel B. Liisberg, Vanessa Rück, Jiro Kondo, Tom Vosch

**Affiliations:** Nanoscience Center and Department of Chemistry, University of Copenhagen Universitetsparken 5 2100 Copenhagen Denmark tom@chem.ku.dk; Department of Materials and Life Sciences, Sophia University 7-1 Kioi-cho Chiyoda-ku 102-8554 Tokyo Japan j.kondo@sophia.ac.jp

## Abstract

The effect of replacing guanosines with inosines in the two stabilizing strands (5′-CACCTAGCGA-3′) of the NIR emissive DNA-Ag_16_NC was investigated. The spectroscopic behavior of the inosine mutants is position-dependent: when the guanosine in position 7 was exchanged, the nanosecond fluorescence decay time shortened, while having the inosine in position 9 made the decay time longer. Thanks to structural information gained from single crystal X-ray diffraction measurements, it was possible to propose a mechanistic origin for the observed changes.

## Abstract 2

The effect of replacing guanosines with inosines in the two stabilizing strands (5′-CACCTAGCGA-3′) of the NIR emissive DNA-Ag_16_NC was investigated.

## Graphical abstract

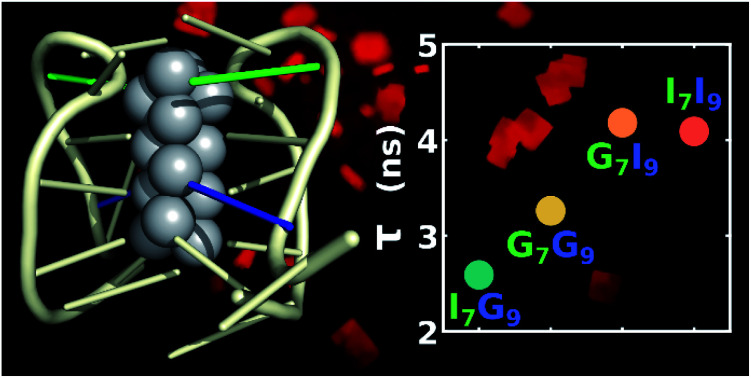

## Introduction

DNA-stabilized silver nanoclusters (DNA-AgNCs) are emissive systems comprising a small aggregate of silver atoms and cations wrapped in one or multiple DNA strands.^1–6^ The spectral properties, fluorescence quantum yield and decay time vary for each DNA-AgNC and depend on variables such as the DNA scaffold and number of reduced silver atoms.^2^ While significant progress has been made in recent years, the relationship between AgNC structure, DNA scaffold and photophysical properties has not been fully elucidated yet. Recently, Zhang *et al.* used transient infrared absorption spectroscopy to investigate the effect of exchanging the only guanosine with inosine in the 18-mer DNA strand (5′-CCCCACCCCTCCCGTTTT-3′) that stabilizes a green emissive DNA-AgNC.^7,8^ The sole difference between guanosine and inosine is the presence or absence of an amino group at the C2 position, which dramatically affected the fluorescence quantum yield and decay time of the green-emitting Ag_10_^6+^ cluster.^8^

Based on these intriguing findings and the apparent critical role of the guanosine on the photophysical properties, we investigated the impact of a similar substitution on the photophysics of a well-characterized NIR emissive DNA-AgNC (further defined as DNA-Ag_16_NC), of which the molecular structure is known.^9–11^ The Ag_16_NC is embedded in two 10-base oligomers (5′-CACCTAGCGA-3′), containing two guanosines. We therefore designed three inosine (I) mutants: I7 (5′-CACCTAICGA-3′) and I9 (5′-CACCTAGCIA-3′), where the guanosine in position 7 and 9 was replaced respectively, and I7–I9 (5′-CACCTAICIA-3′) where both guanosines were substituted. The amino group of guanosine at the C2 position can potentially affect the DNA-Ag_16_NC in two ways. First, its absence can change the hydrogen bonding network between nucleobases and the overall stability of the construct. Secondly, when in close proximity to the AgNC, the amino group can introduce dynamic quenching by photo-induced electron transfer (PET). Single crystal X-ray diffraction measurements unravelled the structure of the I7 and I7–I9 mutants, allowing us to understand how the observed spectroscopic changes are related to the amino groups in position 7 and 9. This was done under the assumption that the mutated positions were the dominant cause of the observed photophysical changes.

## Results and discussion

### Comparison between structure and spectroscopic features

**Fig. 1:**
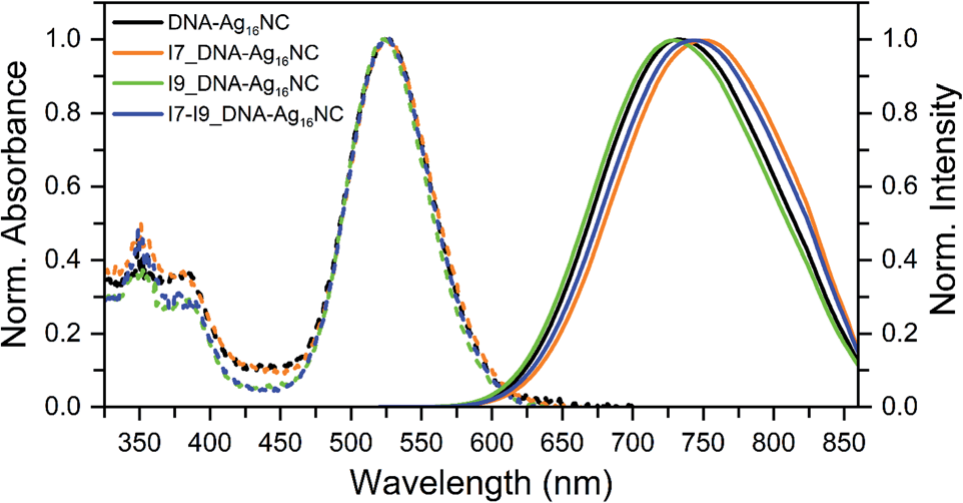
Normalized absorption (at the 525 nm peak) and emission spectra of the original DNA-Ag_16_NC, I7, I9 and I7–I9 mutants, synthesized and measured in a 10 mM ammonium acetate (NH_4_OAc) H_2_O solution at 25 °C. The emission spectra were recorded exciting at 507.5 nm with a picosecond-pulsed laser.

All inosine mutants of DNA-Ag_16_NC were synthesized according to the protocol reported previously.^9^ Details on the synthesis, HPLC purification and the collected fractions can be found in the ESI and Fig. S1.†Fig. 1 shows the normalized absorption and emission spectra of the three inosine mutants, along with the original DNA-Ag_16_NC. The main absorption peak around 525 nm is nearly identical for all four compounds, while the emission spectra display minor shifts in the maximum. The steady-state and time-resolved photophysical values are reported in Table 1.

**Table 1:** Steady-state and time-resolved spectroscopic properties of all inosine-modified DNA-Ag_16_NCs in 10 mM NH_4_OAc H_2_O solution, compared to the original DNA-Ag_16_NC^a^

	*λ*_abs_ (nm)	*λ*_em_ (nm)	*Q* 25 °C	<*τ*_w_> 5 °C (ns)	<*τ*_w_> 25 °C (ns)	<*τ*_w_> 40 °C (ns)	*V*_hydro_ (nm^3^)
DNA-Ag_16_NC	525^b^	736^b^	0.26^b^	3.70^b^^,^^c^	3.26^b^^,^^c^	2.99^b^^,^^c^	10.32^b^^,^^d^
I7_DNA-Ag_16_NC	527	750	0.20	3.28	2.59	2.10	9.53
I9_DNA-Ag_16_NC	524	729	0.36	4.44	4.18	3.94	10.35
I7–I9_DNA-Ag_16_NC	525	744	0.31	4.37	4.09	3.86	10.16

a Absorption maxima (*λ*_abs_), emission maxima (*λ*_em_) and quantum yield (*Q*) at 25 °C, and intensity-weighted average decay time over the whole emission range (<*τ*_w_>) at different temperatures. *Q* was calculated using Cresyl Violet in absolute ethanol as reference dye (0.56).^12^*V*_hydro_ indicates the hydrodynamic volume.

b Data taken from ref. 9.

c Intensity-averaged decay times (<*τ*>) monitored at 730 nm.

d Data taken from ref. 9 and plotted as described in the ESI. The slope of the zero-intercept linear fit is the reported hydrodynamic volume. The data can be found in Fig. S16–S20.

**Fig. 2:**
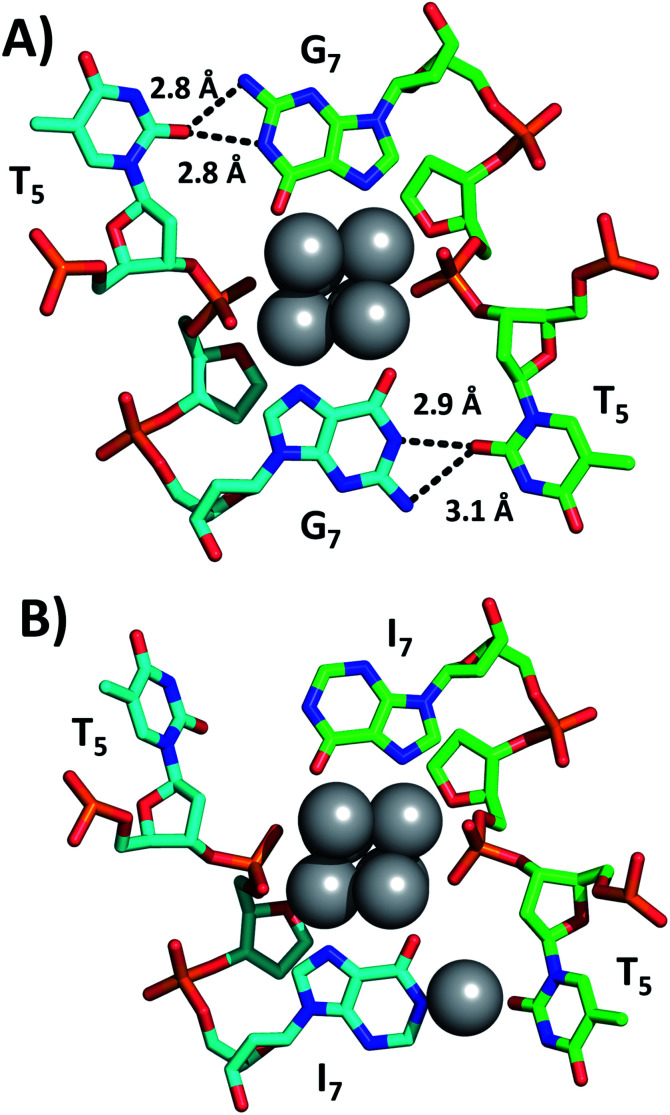
Sections of (A) the original DNA-Ag_16_NC and (B) the I7 mutant, highlighting positions 5 and 7. Selected hydrogen bonds between nucleobases are depicted as black dashed lines, together with the corresponding distances between nitrogen and oxygen atoms.

Fig. 2 shows a section of the original DNA-Ag_16_NC crystal structure (PDB-ID = 6JR4) together with the same region in the I7 mutant (PDB-ID = 7XLV). Details on the crystallization, X-ray measurements, structure determination and photophysical properties of the I7 mutant crystals can be found in the ESI† (see section 4, Table S1 and Fig. S2–S4†).

Emission spectra and decay times of the crystals confirm that the crystal structure is a good proxy for the solution structure. The G_7_ base in DNA-Ag_16_NC coordinates two silvers *via* N7 and O6, while the hydrogen atoms on N1 and N2 of G_7_ can form hydrogen bonds with O2 of T_5_ (2.8–3.1 Å). As shown in Fig. 2B, the I7 mutant lacks the N2 amino group and the increased distance between N1 of I_7_ and O2 of T_5_ indicates that no hydrogen bonds can be formed. Intriguingly, the deprotonated N1 of one of the I_7_ bases is coordinated by Ag with an occupancy of 0.57. This is most likely a silver cation, which interacts at the same time with the oxygen of a water molecule (not shown). It has been observed previously that DNA-AgNCs can contain isolated silver cations in their structures.^10,13^ The lack of hydrogen bonds between I_7_ and T_5_ makes the two T_5_s more flexible, which can explain the drop in the decay time of the I7 mutant with respect to the original DNA-Ag_16_NC (Table 1).^9^ The decrease of <*τ*_w_> to 2.59 ns is similar to other previously reported mutants of DNA-Ag_16_NC, where the T_5_ position replaced with X_5_ (abasic site, PDB-ID = 7BSH), A_5_ (adenosine, PDB-ID = 7BSE) and G_5_ (guanosine, G5-NIR mutant PDB-ID = 7BSG). Their average decay times were 2.42, 2.54, and 2.52 ns, respectively.^15^ Interestingly, the absence of O2 in the fifth nucleobase of X5, A5 and G5-NIR mutants also prevents the formation of hydrogen bonds with the G7 nucleobases (see Fig. S5†), whereas the C5 mutant (cytosine, PDB-ID = 7BSF) can still form hydrogen bonds with G_7_s, resulting in a <*τ*_w_> of 3.35 ns that is similar to the original DNA-Ag_16_NC (see Fig. S5†). These observations point towards the importance of hydrogen bonds between position 5 and 7 on <*τ*_w_>.

**Fig. 3:**
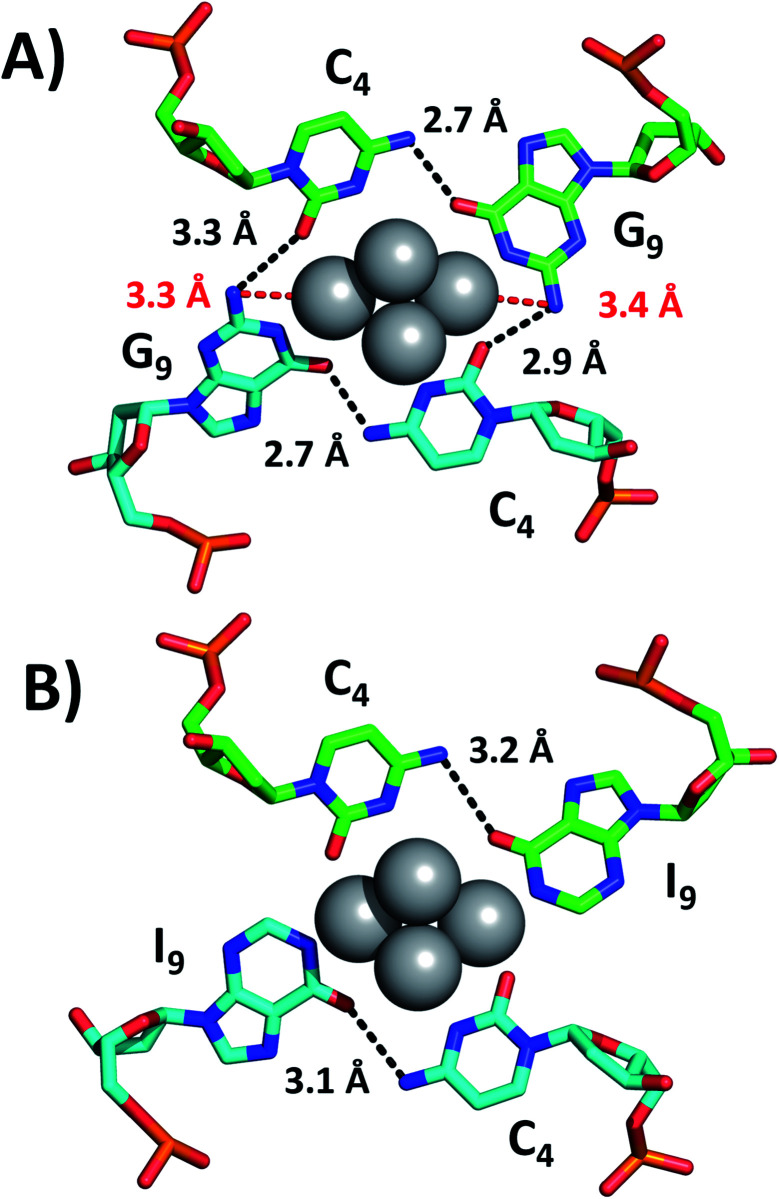
Positions 4 and 9 of (A) the original DNA-Ag_16_NC and (B) the I7–I9 mutant crystals. The distances between N2 in G_9_s and the nearest silver atoms are represented as red dashed lines, whereas selected hydrogen bonds are depicted as black dashed lines.

While we were unable to determine the structure of the I9 mutant, we managed to crystallize and resolve the structure of the I7–I9 double mutant. Therefore, we used the structural information obtained from the I7–I9_DNA-Ag_16_NC crystal around position 9 and assumed that it can provide a reasonable picture of the spectroscopic behavior of the I9 mutant. Details on the single crystal X-ray diffraction measurements and analysis, along with the photophysical properties of I7–I9 mutant crystals can be found in the ESI† (section 4, Table S1 and Fig. S6–S8†). Fig. 3 shows the section involving positions 4 and 9 for both the original DNA-Ag_16_NC and the I7–I9 mutant (PDB-ID = 7XLW). G9 interacts with Ag through O6 and the deprotonated N1. Additional stabilization is ensured by hydrogen bonds between G_9_ and C_4_, particularly between O6 (G_9_) and the exocyclic N4 (C_4_) and potentially between N2 (G_9_) and O2 (C_4_), although the latter distances are larger (2.9–3.3 Å).^10^ Consequently, when G_9_ is replaced by I_9_, the two potential hydrogen bonds between N2(G_9_)–O2(C_4_) are missing. Since the I_9_ and C_4_ nucleotides are directly bound to the Ag_16_NC, the absence of these possible interactions might be less relevant or even not relevant at all. Note that there are six I7–I9_DNA-Ag_16_NCs in the asymmetric unit cell of the I7–I9 mutant (see Fig. S23†) with O6(I_9_)–N4(C_4_) distances spanning from 2.5 to 3.2 Å. The distances from N2 in the G_9_s to the nearest silver atoms are 3.3–3.4 Å (red dashed lines in Fig. 3A). While too far for creating coordination bonds,^10,16^ they are still plausible distances for PET.^17^ This could be the reason for the increase of <*τ*_w_> and *Q* in the I9 mutant, where the amino groups are removed and hence potential dynamic quenching is eliminated. Note that the changes in <*τ*_w_> and *Q* are percentage-wise similar, supporting the idea of dynamic quenching. Interestingly, some of the A_10_ nucleotides point towards the AgNC, while others point away in order to promote crystal packing interactions (Fig. S23†).

The space group of I7–I9 mutant crystal is *P*1 (*i.e.* triclinic) with a very intricate unit cell (Fig. S23†), while the I7 mutant crystal is orthorhombic (*P*2_1_2_1_2_1_) with one I7_DNA-Ag_16_NC per unit cell (Fig. S22†). When only one guanosine is replaced, I7 and I9 appear to shorten and lengthen <*τ*_w_>, respectively. When both positions are substituted, an average decay time of 4.09 ns is found, which is similar to I9. Fig. S9† shows that hydrogen bonding interactions are still possible between I_7_ and T_5_, making the absence of the amino group in I9 the dominant effect. Note that the low occupancy silver cation bound to I_7_, seen in the I7 mutant, is now absent. The trend of the changes in <*τ*_w_> is also reflected in the variations of *Q*, since both vibration-mediated decay processes and PET are additional decay pathways for the fluorescent state, affecting <*τ*_w_> and *Q* in a similar fashion. As such, DNA-Ag_16_NC and its mutants behave differently from another NIR emissive DNA-AgNC, where we demonstrated that there is no direct relationship between <*τ*_w_> and *Q*, indicating that the changes in *Q* are not directly linked to the emissive state (*e.g.*, in the case of static quenching).^14^

### Luminescence characterization

Recently, we reported that DNA-Ag_16_NC has a long-lived luminescent state when dissolved in a 10 mM NH_4_OAc D_2_O solution.^11^ This luminescence is red-shifted, but at 25 °C it overlaps with the fluorescence and appears as one broad band in the steady-state emission spectrum.

The exact origin of this microsecond state has not been determined yet, but its appearance in D_2_O might indicate the importance of the hydrogen bonding network.^11^

The removal of the amino groups in the inosine mutants does not change the effect of D_2_O reported for the original DNA-Ag_16_NC. When the inosine mutants are dissolved in D_2_O, the fluorescence intensity-averaged decay times, <*τ*_w_>, shorten and microsecond-lived luminescence appears (Table 2). Interestingly, the microsecond luminescence decay time <*τ*_μs_> is lower for the I7 and I7–I9 mutants, while it is unaffected for the I9 modification, indicating that position 7 plays a more relevant role for <*τ*_μs_>. The long-lived state is particularly evident when the inosine mutants are frozen in liquid N_2_: fluorescence peaks blue-shift to 670–680 nm and the long-lived luminescence bands arise around 820–835 nm (Fig. S11† and Table 2). Again, these findings are similar to the original DNA-Ag_16_NC. Decay curves monitored at 820 nm in liquid N_2_ (Fig. S12†) were tail-fitted bi-exponentially resulting in average decay times <*τ*_μs_> of 410–436 μs. At 25 °C, values between 45 and 85 μs were found (Fig. S10†). The presence of long-lived states was also confirmed by time-correlated single photon counting (TCSPC) measurements at −196 °C. As reported in ref. 11 and 20, the background amplitude counts can be used as a proxy for creating the red-shifted microsecond-lived luminescence spectrum (Fig. S13–S15†).^11^ Time-resolved emission spectra (TRES), constructed from the ns decay curves recorded at different wavelengths, yielded spectra similar to the original DNA-Ag_16_NC.^11^

**Table 2:** Steady-state and time-resolved spectroscopic properties of the original DNA-Ag_16_NC and the inosine mutants synthesized in a 10 mM NH_4_OAc H_2_O solution and measured in a 10 mM NH_4_OAc D_2_O solution^a^

	25 °C			−196 °C			
	*Q*	<*τ*_w_> (ns)	<*τ*_μs_> (μs)	*λ*_fluo_ (nm)	<*τ*_w_> (ns)	*λ*_μs_ (nm)	<*τ*_μs_> (μs)
DNA-Ag_16_NC	0.18^b^	2.21^b^^,^^c^	79	682	2.12^b^^,^^d^^,^^e^	840	447^b^^,^^e^^,^^f^
I7_DNA-Ag_16_NC	0.15	1.99	45	680	2.09	834	436
I9_DNA-Ag_16_NC	0.23	2.46	85	671	2.03	820	421
I7–I9_DNA-Ag_16_NC	0.21	2.46	50	678	2.06	828	410

a *Q* and <*τ*_w_> are the fluorescence quantum yield and intensity-weighted average decay time over the whole emission range, respectively. *Q* was calculated with a relative method (see Fig. S21).^12^*λ*_fluo_ and *λ*_μs_ define, respectively, the fluorescence and luminescence emission maxima, measured on a FluoTime300 instrument. <*τ*_μs_> is the microsecond average decay time obtained by tail-fitting the decay curves monitored at 820 nm (Fig. S10 and S12).

b Data taken from ref. 11.

c Decay monitored at 740 nm.

d Decay monitored at 720 nm.

e DNA-Ag_16_NCs synthesized and measured in a 10 mM NH_4_OAc D_2_O solution.

f Decay monitored at 810 nm.

**Fig. 4:**
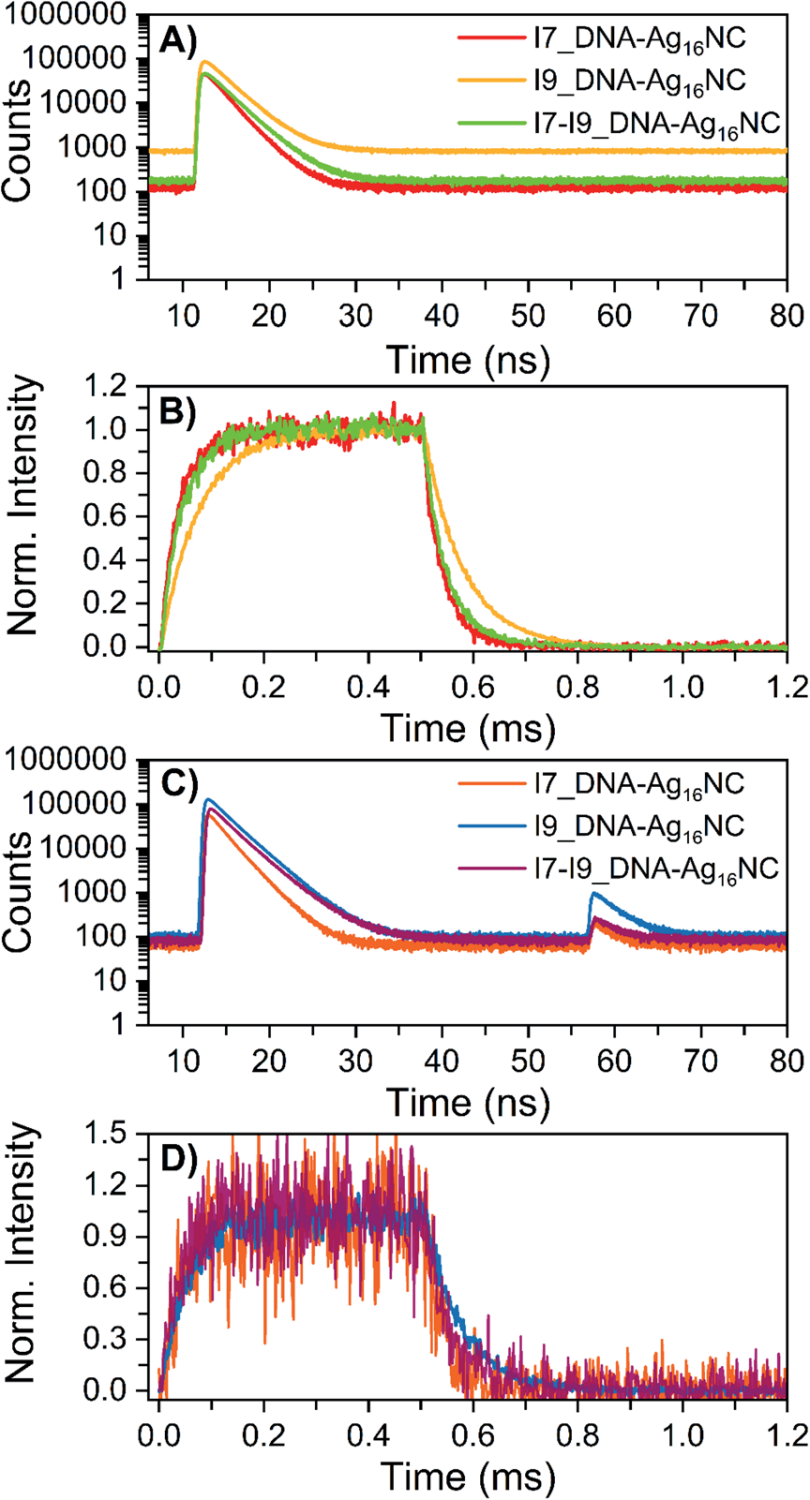
Micro-time decay curves and temporal evolution of the primary fluorescence, luminescence and OADF (optically-activated delayed fluorescence) in the macro-time domain for the inosine mutants in 10 mM NH_4_OAc D_2_O solutions, measured on a home-built confocal setup at room temperature. (A) Nanosecond decay curves (24 W cm^−2^; *f*_Micro_ = 11 MHz, *f*_Macro_ = 500 Hz, *T*_on_ = 0.5 ms and *T*_off_ = 1.5 ms), constructed from the micro-times. (B) Temporal evolution of the luminescence based on the gate in the micro-time domain below 10 ns and above 40 ns, exciting at 520 nm in a burst mode setting (24 W cm^−2^; *f*_Micro_ = 11 MHz, *f*_Macro_ = 500 Hz, *T*_on_ = 0.5 ms and *T*_off_ = 1.5 ms). (C) Primary and secondary fluorescence decays recorded during 520 nm (632 W cm^−2^; *f*_Micro_ = 11 MHz, *f*_Macro_ = 500 Hz, *T*_on_ = 0.5 ms, and *T*_off_ = 1.5 ms) and 850 nm (6.5 kW cm^−2^; *f*_Micro_ = 11 MHz, *f*_Macro_ = 500 Hz, and *T*_on_ = 2 ms) co-illumination in a burst mode setting. (D) Photons that were part of the secondary fluorescence decays between 55 and 75 ns were utilized to gate the OADF contribution in the macro-time domain. Details on the method and data analysis are reported in section 3.3 of ESI† and ref. 18.

The microsecond-lived state was further investigated for all inosine mutants by using a burst mode approach, where every detected photon is associated with a micro- and a macro-time (see ESI† for details).^18^ The micro-times (Fig. 4A and C) can be used to gate different contributions to the overall emission in the macro-time domain, as shown in Fig. 4B and D. During the time the pulsed 520 nm laser is on, luminescence from the long-lived state rises and reaches a steady-state equilibrium. When the laser is switched off at 0.5 ms, the luminescence decays and this part can be used to determine <*τ*_μs_>. The obtained <*τ*_μs_> are reported in Table 3 and are in line with the values in Table 2 recorded with a Xe flash lamp (repetition rate = 300 Hz).

**Table 3:** Microsecond decay times of the luminescent state (<*τ*_μs_>) and OADF (<*τ*_OADF_>) process for all inosine-modified DNA-Ag_16_NCs synthesized in a 10 mM NH_4_OAc H_2_O solution and measured in a 10 mM NH_4_OAc D_2_O solution at room temperature^a^

	<*τ*_μs_> (μs)	<*τ*_OADF_> (μs)	OADF efficiency
I7_DNA-Ag_16_NC	39	35	0.23%
I9_DNA-Ag_16_NC	75	70	0.66%
I7–I9_DNA-Ag_16_NC	46	50	0.22%

a The time-resolved values were obtained by tail-fitting mono-exponentially the decays shown in Fig. 4B and D. Details on the measurements are reported in section 3.3 of the ESI. The OADF efficiency is defined as the secondary fluorescence divided by the primary fluorescence.

Recently, we have proven for DNA-Ag_16_NC that the luminescent state is capable of generating optically-activated delayed fluorescence (OADF)^18^ when the primary 520 nm laser is combined with a delayed secondary NIR laser (850 nm). Note that 850 nm was chosen since it was used in a previous publication,^18^ but it might not be the most efficient wavelength.^19^ As shown in Fig. 4C, the secondary fluorescence decays appear in the micro-time domain after 55 ns. This OADF micro-time information can be used again as a gate to reconstruct the temporal evolution of the OADF in the macro-time domain (Fig. 4D). Like the original DNA-Ag_16_NC, the OADF decay times (Table 3) of the inosine mutants match the decay time values of the luminescent state, indicating that the latter is responsible for the OADF process. Minor variations in the OADF efficiency can be observed, with the I9 mutant characterized by the highest value under these experimental conditions. This could simply be because the I9 mutant has the longest <*τ*_μs_>.

## Conclusions

In summary, we designed three mutants where guanosines were substituted with inosines in the two stabilizing strands of DNA-Ag_16_NC. The resulting photophysical behavior was found to be position-dependent. When position 7 was replaced, the absence of the amino groups resulted in the lack of hydrogen bonds with T_5_, which made the construct less rigid. This could be the reason for the shortening of <*τ*_w_> and the decrease of *Q* by increased contribution from vibration-mediated non-radiative decay pathways. A similar drop in <*τ*_w_> was observed for three other DNA-Ag_16_NC mutants, where position 5 was exchanged to either an abasic site or a purine. Our results indicate that, while the base in position 5 was not directly bound to the AgNC, the hydrogen bonding interactions with it have an important role for the overall flexibility of the construct. Replacing the guanosine in position 9 produced an opposite effect: <*τ*_w_> and *Q* increased, which could be due to the absence of a dynamic type quenching by photo-induced electron transfer from the amino groups. Interestingly, the doubly substituted mutant had spectroscopic features more similar to the I9 mutation, with respect to <*τ*_w_>, while it was more alike to the I7 mutant in relation to <*τ*_μs_>. We note that the effect of replacing guanosine with inosine is significantly larger for the green Ag_10_^6+^ emitter from Petty *et al.*^7^ Unfortunately, no crystal structure is currently available for this green emitter to make comparisons with our work.

Like the original DNA-Ag_16_NC, the inosine mutants showed microsecond-lived states that were probed with multiple approaches: TCSPC, the use of a Xe flash lamp and a novel burst mode method, together with the ability to generate OADF. <*τ*_μs_> and <*τ*_OADF_> were found to be akin, suggesting that also for the inosine mutants, the luminescent state is responsible for the OADF process.

## Author contributions

C. C. and V. R. synthesized and performed the spectroscopic measurements of the inosine-modified DNA-Ag_16_NCs. M. B. L. performed the burst-mode measurements. J. K. crystallized the inosine mutants and carried out the single crystal X-ray diffraction measurements and data analysis. C. C. and T. V. conceived the experiments. The paper was written with input from all authors.

## Conflicts of interest

There are no conflicts to declare.

## Supplementary Material

NA-004-D2NA00325B-s001
