# Supplementary material for: The effect of inosine on the spectroscopic properties and crystal structure of a NIR-emitting DNA-stabilized silver nanocluster
Source: Nanoscale Adv. 2022 Jun 20;4(15):3212–7. doi: 10.1039/d2na00325b (PMC9416947; doi:10.1039/d2na00325b)
Supplement: NA-004-D2NA00325B-s001 [file NA-004-D2NA00325B-s001.pdf]

Electronic Supplementary Material (ESI) for Nanoscale Advances.
This journal is © The Royal Society of Chemistry 2022

Supporting Information

The effect of inosine on the spectroscopic properties and crystal structure of a NIR-emitting DNA-stabilized silver nanocluster.

Cecilia Cerretani,[a] Mikkel B. Liisberg,[a] Vanessa Rück,[a] Jiro Kondo[b,]* and Tom Vosch[a,]*

[a] *Nanoscience Center and Department of Chemistry, University of Copenhagen, Universitetsparken 5, 2100 Copenhagen, Denmark. E-mail: tom@chem.ku.dk.*
[b] *Department of Materials and Life Sciences, Sophia University, 7-1 Kioi-cho, Chiyoda-ku, 102-8554 Tokyo, Japan. E-mail: j.kondo@sophia.ac.jp.*

MATERIALS AND METHODS

1. Inosine-modified DNA-Ag_{16}NCs synthesis

All oligonucleotides (5'–CACCTAICGA–3', 5'–CACCTAGCIA–3', and 5'–CACCTAICIA–3') were purchased from Integrated DNA Technologies (IDT). Silver nitrate ($AgNO_3$, ≥99.998%) and sodium borohydride ($NaBH_4$, 99.99%) were purchased from Sigma Aldrich. All chemicals were used as received. All solutions were prepared in nuclease-free water (IDT).

The DNA-AgNCs were synthesized by mixing the hydrated DNA with $AgNO_3$ in a 10 mM ammonium acetate (NH_4OAc) aqueous solution at pH 7. After 15 minutes, $NaBH_4$ was added in order to reduce the silver cations. Like the original DNA-Ag_{16}NC, the final ratio of the components was [DNA]:[Ag^+]:[BH_4^-] = 25 µM: 187.5 µM: 93.75 µM.[1]

After storing the samples at 4 ^{o}C for three days, HPLC purification was performed, and the collected fractions were finally solvent-exchanged to 10 mM NH_4OAc H_2O solution by spin-filtration (cut-off membrane = 3 kDa).

2. High-performance liquid chromatography (HPLC) purification

The HPLC purification was performed using a preparative HPLC system from Agilent Technologies with an Agilent Technologies 1260 Infinity fluorescence detector, an Agilent Technologies 1100 Series UV-Vis detector and a Kinetex C18 column (5 µm, 100 Å, 250 × 4.6 mm, Phenomenex), equipped with a fraction collector. The mobile phase was a gradient mixture of 35 mM triethylammonium acetate (TEAA) buffer in MilliQ water (A) and methanol (B).

The same HPLC method was used for all inosine modifications. The gradient was varied from 20% to 95% B as follows: 0-2 min 20% B, 2-27 min linear increase of B until 45%, 27-30 min from 45% to 95% B. The run was followed by 5 min of washing with 95% B to remove any traces of the sample from the column. The flow rate was set to 1 mL/min, and the fraction collection was based on the absorption signal at 530 nm.

As shown in the chromatograms in Figure S1, the retention time was different for the three mutants: I9_DNA-Ag_{16}NCs eluted at 16 min (≈34% B), I7_DNA-Ag_{16}NCs were collected at 20 min (≈38% B), while I7-I9_DNA-Ag_{16}NCs eluted around 22.5 min (≈40-41% B).

The HPLC method was slightly modified compared to the original DNA-Ag_{16}NC purification method.[1]

3. Spectroscopic measurements

Steady-state and time-resolved measurements were performed in both 10 mM NH_4OAc H_2O and D_2O solutions at diverse temperatures: -196, 5, 25, and 40 ^{o}C. Measurements at -196 ^{o}C will be described in paragraph 3.2.4.

3.1 *Absorption measurements*

Absorption spectra were measured with a Cary 300 UV-Vis spectrophotometer from Agilent Technologies using a deuterium lamp for ultraviolet radiation and a tungsten-halogen lamp for visible and near-infrared (NIR) radiation.

3.2 FluoTime300 Instrument

3.2.1 Steady-state emission measurements

Steady-state fluorescence measurements were performed using a FluoTime300 instrument from PicoQuant. Emission spectra were recorded with picosecond-pulsed lasers (PicoQuant): 531 nm (LDH-D-TA-530B), 507.5 nm (LDH-P-C-510), or 532 nm (VisUV-266-355-532 multicolor module), and then corrected for the wavelength dependency of the detector.

3.2.2 Quantum yield measurements and calculations

Quantum yield (Q) values for the inosine mutants were determined at 25 ^{0}C in both 10 mM NH_4OAc H_2O and D_2O solutions, using Cresyl Violet in absolute ethanol as a reference (Q=0.56).[2] Absorption and emission spectra of the inosine mutants and the reference compound were measured at different concentrations in order to calculate the corresponding Q, according to the following formula[2]:

$$\boldsymbol{Q_{NC}} = \frac{F_{NC}}{f_{A,NC}} \cdot \frac{f_{A,ref}}{F_{ref}} \cdot \frac{n_{NC}^2}{n_{ref}^2} \cdot \boldsymbol{Q_{ref}} \quad \textbf{(1)}$$

where ***Q*** represents the quantum yield, ***F*** is the integrated emission spectrum (*i.e.,* the area under the fluorescence spectrum), $\boldsymbol{f_A}$ defines the fraction of absorbed light at the excitation wavelength (532 nm), and ***n*** is the refractive index of the medium where the compounds are dissolved in during the measurements. Changes in the refractive index due to isotope variations are ignored since they are in the 1% difference range. The subscripts ***NC*** and ***ref*** indicate the DNA-AgNC and the reference dye, respectively.

3.2.3 Time-correlated single photon counting (TCSPC) measurements and data analysis

Time-resolved fluorescence and anisotropy measurements were performed exciting with a vertically-polarized (V) 507.5 nm pulsed laser at three different temperatures: 5, 25, and 40 ^{0}C. Fluorescence decay curves were measured every 5 nm in the 630 - 830 nm range. The integration time was varied between 15 and 40 s, whereas the laser repetition rate was selected from 10.0 to 20.0 MHz in order to reach at least 10,000 counts in the maximum at λ_{em}^{max}.[3]

The analysis of time-resolved data was performed with FluoFit v.4.6 software from PicoQuant. All decay curves were globally fitted with a mono- or multi-exponential reconvolution model including the instrument response function (IRF). The fluorescence intensities at three discrete times after the excitation pulse (0.1, 1, 10 ns) were plotted as a function of emission wavelength in order to construct time-resolved emission spectra (TRES). TRES were then corrected for the detector efficiency and interpolated with a spline function using the built-in *spaps* MATLAB function with a tolerance of 10^{-10} (*i.e.*, forcing the interpolated curve to go through the data points).

Time-resolved anisotropy measurements were carried out by recording parallel (VV) and perpendicular (VH) fluorescence decays at λ_{em}=730 nm. In order to reach at least 10,000 counts

in the maximum, the integration time was varied between 15 and 60 s, while the laser repetition rate ranged from 10.7 to 20.0 MHz. The decay curves were fitted with FluoFit v.4.6 from PicoQuant using, respectively, a multi-exponential and a mono-exponential reconvolution model for the decay time and the rotational correlation time (θ), including the IRF.

Based on the Perrin equation[3], $\boldsymbol{\theta} = \frac{\eta V_{hydro}}{k_B T}$, θ values were plotted as a function of $\boldsymbol{\eta / k_B T}$, where $\boldsymbol{\eta}$ is the dynamic viscosity of the solvent, $\boldsymbol{k_B}$ is the Boltzmann constant and $\boldsymbol{T}$ is the absolute temperature. The data was fitted linearly while fixing the y-intercept at zero. The resulting slope was the hydrodynamic volume ($\boldsymbol{V_{hydro}}$) of the clusters (assumed to be spherical).

3.2.4 Steady-state and fluorescence decay measurements at -196 ^{o}C

Low temperature steady-state and time-resolved emission measurements were carried out only in a 10 mM NH_4OAc D_2O solution by immersing a NMR tube with the sample in a transparent Dewar filled with liquid nitrogen (-196 °C). The Dewar was then placed in the cuvette holder of a FluoTime300 instrument from PicoQuant. In order to limit the large scattering, due to the presence of ice, two filters were used: a 510 nm band-pass filter (Semrock, FF02-510/10-25) in the excitation path and a 532 nm long-pass (Semrock, BLP01-532R-25) in the emission path.
Fluorescence intensity decays, as well as steady-state emission spectra, were measured exciting with a 507.5 nm pulsed laser. Emission spectra were then corrected for the detector efficiency.
Fluorescence decay curves were measured every 5 nm in the 610 - 850 nm range. The integration time was set to 10 s, whereas the laser repetition rate was selected between 13.3 and 20.0 MHz in order to reach at least 10,000 counts in the maximum at λ_{em}^{max}. The data analysis was carried out with FluoFit v.4.6 software (PicoQuant). The decays were globally fitted with a tri-exponential reconvolution model including the IRF. The amplitude (α_i) and decay time (τ_i) components were used to calculate the intensity-averaged decay time, $\langle\tau\rangle$,[3] and the corresponding intensity as a function of emission wavelength. $\langle\tau_w\rangle$, *i.e.,* the overall intensity-weighted average decay time, was then calculated as the average of $\langle\tau\rangle$ over the emission spectra weighted by the steady-state intensity. Furthermore, the background amplitude of the decays (*Bkgr*) was plotted as a proxy for the microsecond-lived emission. Both the background amplitude and the intensity of $\langle\tau\rangle$ were corrected for the detector efficiency. Finally, the steady state intensity (*I*) was calculated by using the following formula:

$$\boldsymbol{I = Bkgr + \sum_{\lambda}(\sum_i \alpha_i \tau_i)_{\lambda}} \quad \textbf{(2)}$$

3.2.5 Microsecond decay measurements

Microsecond decays were acquired for all inosine mutants in a 10 mM NH_4OAc D_2O solution at 25 and -196 ^{o}C. Details on the low temperature measurements are reported in the previous paragraph. The samples were excited at 508 nm with a Xenon flash lamp (repetition rate = 300 Hz), and the decay curves were recorded at 820 nm with an integration time of 10 min. The decays at 25 ^{o}C (Figure S10) and -196 ^{o}C (Figure S12) were tail-fitted with a mono- and bi-exponential function, respectively, with FluoFit v.4.6 software from PicoQuant. The microsecond decay times, $\langle\tau_{\mu s}\rangle$, can be found in Table 2.

3.3 Home-built microscope setup

Nanosecond fluorescence and long-lived luminescence of inosine-modified DNA-Ag_{16}NCs were simultaneously measured on our home-built confocal microscope in a burst mode approach described in detail previously.[4] In brief, the method relies on exciting the sample with micro- to millisecond bursts of a high repetition rate excitation light source, which, within its on-time, builds up a population of long-lived luminescent states that decays without interfering with fluorescence, once the excitation source is turned off. At the same time, this approach allows to determine the nanosecond-lived fluorescence. This cycle is repeated until sufficient photons are collected to construct the nano- and micro-second decay curves. By using diverse gating schemes, it is possible to extract pure fluorescent and long-lived luminescent photons.[4] Co-illumination measurements were additionally performed to investigate the optically activated delayed fluorescence (OADF) contribution of the DNA-AgNCs. A description of the optical setups used for single and co-illumination measurements will follow.

3.3.1 Single wavelength excitation setup

For single wavelength measurements, a fiber coupled (FD7-PM, NKT Photonics) pulsed 11 MHz continuum white-light laser (SuperK EXTREME EXB-6, NKT Photonics) was used as an excitation source delivering a wavelength of 520 nm by sending the continuum output through an acousto-optic tunable filter (AOTF; SuperK SELECT, NKT Photonics). The output of the fiber was expanded and collimated by a lens system and cleaned up by a 520 nm band-pass filter (FF01-520/5-25, Semrock) and a 561 nm short-pass filter (SP01-561RU-25, Semrock). Then it was reflected by a 30:70 beam splitter (XF122, Omega Optical) and sent through an air objective (CPlanFLN 10x, NA = 0.3, Olympus), which focused the laser onto the sample and collected the luminescence. The laser light was blocked by a 561 nm long-pass filter (BLP01-561R-25, Semrock) and out-of-focus light was blocked by a 100 µm pinhole. To increase the long-lived luminescence contribution relative to the fluorescence, an 850 nm band-pass filter (FF01-850/10-25, Semrock) was inserted in the emission path. The resulting emission was detected on an avalanche photodiode (CD3226, PerkinElmer) connected to a single photon counting module (SPC-830, Becker & Hickl).

3.3.2 Co-Illumination Setup

For co-illumination measurements, a second excitation path of 850 nm was introduced. The AOTF has two crystals, one for producing visible wavelengths and another for near infrared (NIR) wavelengths. Thus, the 850 nm output from the NIR port of the AOTF was cleaned with an 850 nm band-pass filter (FF01-850/10-25, Semrock) and directed towards the primary excitation beam. The two beams (520 nm and 850 nm) were combined with a dichroic mirror (TLP01-501-25x36, Semrock) and subsequently reflected by a second dichroic mirror (TLP01-628-25x36, Semrock) and sent through an oil immersion objective (UPlanSApo 100x, NA = 1.4, Olympus) that focused the laser onto the sample and collected the luminescence. Compared to the single wavelength excitation setup, a 750 nm short-pass filter was used in the emission path, replacing the 850 nm band-pass filter (FF01-850/10-25, Semrock). The 520 nm beam went through an optical fiber. Due to the optical path length differences between the two beams, the secondary pulse appears 45 ns delayed with respect to the primary.

4. Crystallization

4.1 Crystal growth

Crystals of I7 and I7-I9 mutants were grown in an incubator at 293 K by the hanging-drop vapor-diffusion method. 0.2 µL of cluster solution was mixed with 0.2 µL of crystallization buffer and equilibrated against 250 µL of a reservoir solution (either 40% 2-methyl-2,4-pentanediol (MPD) or 40% polyethylene glycol (PEG) 3350). The crystallization buffer contains either 10% MPD or 10% PEG 3350, XNO_3 salt (where X can be Li^+, Na^+, K^+, NH_4^+, Mg^{2+}, Ca^{2+} or Sr^{2+}) with different concentrations, 10 mM spermine and 50 mM 3-(N-morpholino)propanesulfonic acid (MOPS) at pH 7. Crystals were obtained with various conditions after several weeks. The crystallization conditions used for X-ray diffraction measurements and spectroscopic characterization are the following:

- **I7_DNA-Ag_{16}NC**: MPD with 10 mM $LiNO_3$ for X-ray data collection, and MPD with 10 mM, 100 mM and 200 mM $LiNO_3$ for emission and decay time measurements.
- **I7-I9_DNA-Ag_{16}NC**: MPD with 10 mM NH_4NO_3 for X-ray diffraction measurements, PEG 3350 with 10 mM NH_4NO_3 and PEG 3350 with 10 mM $Sr(NO_3)_2$ for spectroscopic characterization.

4.2 Single crystal X-ray diffraction measurements and analysis

4.2.1 X-Ray data collection

Single crystal X-ray diffraction measurements were carried out at 100 K with the synchrotron radiation at the BL-17A beamline in the Photon Factory (Tsukuba, Japan) facility. An X-ray beam with a wavelength of 0.90 or 0.98 Å was used for the data collection. The data sets were recorded using 1° oscillation with 0.1 s exposure per frame. This collection time was found to be optimal, since no significant radiation damage was observed for the crystals.

4.2.2 Structure determination and refinement

The data sets were processed by the program XDS.[5] The initial phases were determined with AutoMR from the Phenix suite[6-8] by molecular replacement using the original DNA-Ag_{16}NC structure as a model (PDB-ID = 6JR4). Molecular models were then constructed by using the program Coot.[9, 10] The atomic parameters were refined by the program phenix.refine of the Phenix suite[11] at maximum resolutions of 1.1 and 1.9 Å, for I7_DNA-Ag_{16}NC and the I7-I9 mutant, respectively. Statistics of data collection and structure refinement are summarized in Table S1.

4.3 Spectroscopic characterization

Bright-field and fluorescence images were recorded on an inverted Olympus IX71 microscope. For the I7 mutant crystals, a 10x objective (CPlanFL N 10x, NA = 0.3, Olympus) was used, while a 20x objective (LCAch N 20x, NA = 0.40, Olympus) was employed for I7-I9_DNA-Ag_{16}NC. For the bright-field images, white light was used. For the fluorescence images, an X-Cite Series 120Q light source was utilized in combination with an Olympus BP510-550 excitation filter, Olympus BA590 emission filter and Semrock FF580-FDi01 dichroic filter. All images (Figures S2 and S6) were recorded with the camera of an iPhone SE.

Fluorescence decay time measurements of individual crystals (Figures S4 and S8) were carried out on our home-built setup identical to that described in section 3.3.1 with the exception of the objective, which was exchanged to a 40x objective (LUCPlanFL N 40x, NA = 0.60, Olympus). All fluorescence decays were tail-fitted with a bi-exponential model with Origin 2020 software. Steady-state emission spectra of individual crystals (Figures S3 and S7) were recorded on the same setup, but the emission was instead redirected through a spectrograph (SP 2356 spectrometer, 300 grooves/mm, Acton Research) onto a nitrogen cooled CCD camera (SPEC-10:100B/LN-eXcelon, Princeton Instruments). The emission spectra were wavelength- and intensity-calibrated as previously described.[12]

HPLC Chromatograms

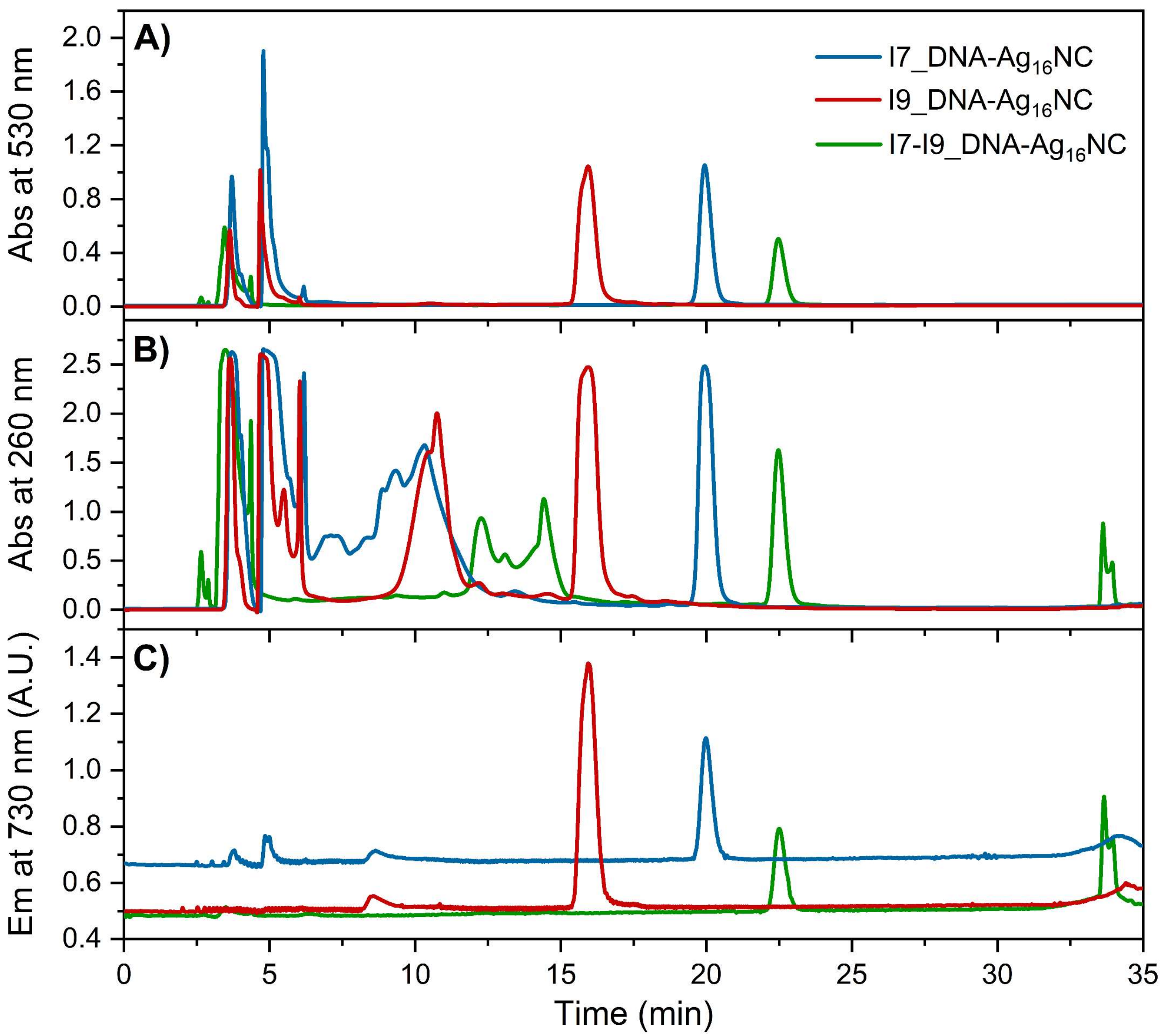

Figure S1. HPLC chromatograms of inosine-modified DNA-Ag_{16}NCs **A)** monitoring the main absorption peak of the clusters at 530 nm, and **B)** monitoring the DNA absorption at 260 nm and **C)** monitoring the emission of the clusters at 730 nm (λ_{exc}= 530 nm). The blue, red, and green traces refer, respectively, to I7_DNA-Ag_{16}NC, I9_DNA-Ag_{16}NC and I7-I9_DNA-Ag_{16}NC, whose elution times are reported in section 2.

I7 Crystals

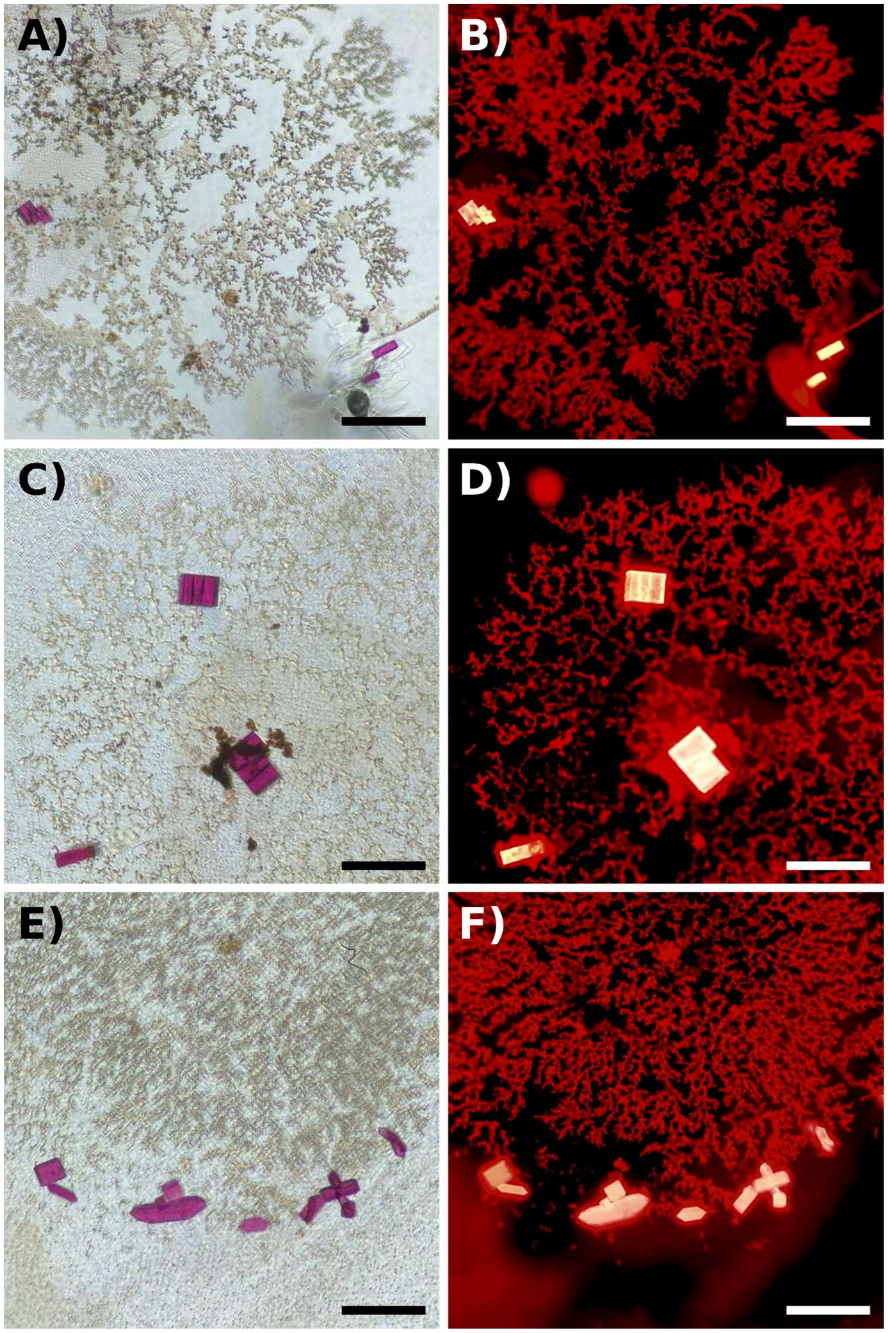

<u>Figure S2.</u> **A, C, E)** Bright-field and **B, D, F)** fluorescence images of I7_DNA-Ag_{16}NC crystals at 10x magnification. The fluorescence intensity of the crystals saturated the intensity of the camera (white color). Images were taken approximately 5 months after starting the crystallization. The crystals were grown with 10% MPD, 10 mM spermine, 50 mM MOPS and **A, B)** 10 mM $LiNO_3$, **C, D)** 100 mM $LiNO_3$ or **E, F)** 200 mM $LiNO_3$. Scalebar: 100 µm.

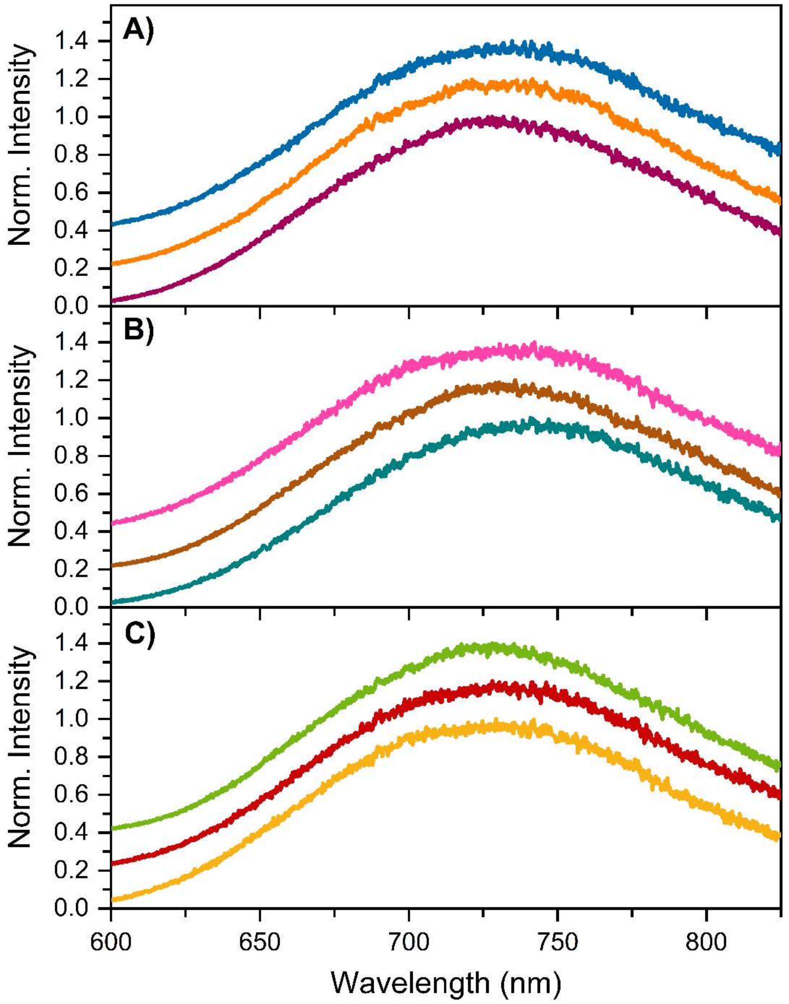

Figure S3. Emission spectra of I7_DNA-Ag_{16}NC crystals, recorded with a confocal microscope upon 520 nm excitation. The spectra are normalized to the emission maximum and have a constant 0.2 offset for displaying purposes. The crystals were grown with 10% MPD, 10 mM spermine, 50 mM MOPS and **A)** 10 mM $LiNO_3$, **B)** 100 mM $LiNO_3$ or **C)** 200 mM $LiNO_3$.

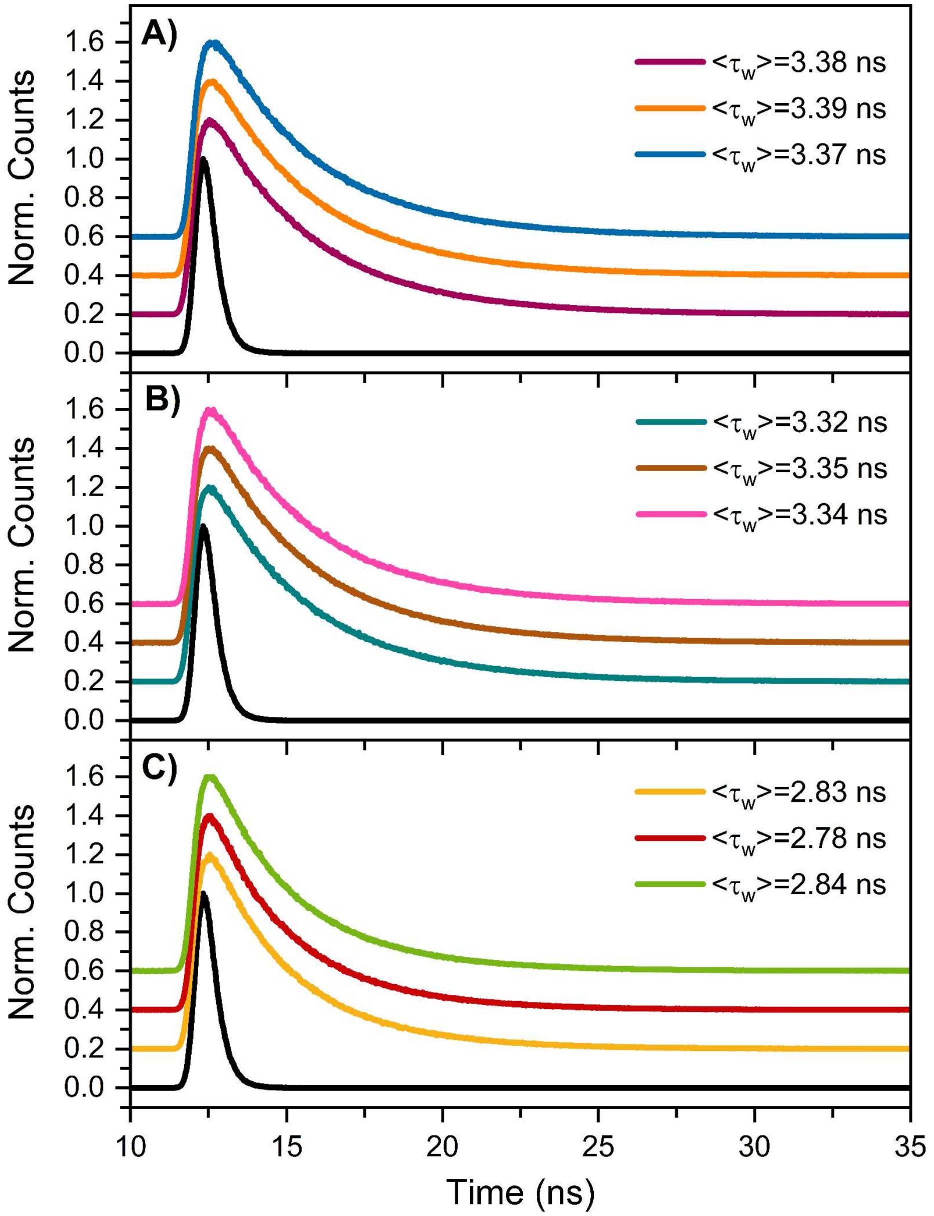

<u>Figure S4.</u> Fluorescence decay curves of I7_DNA-Ag_{16}NC crystals, exciting at 520 nm. The colored curves are the fluorescence decays, while the black curve is the instrument response function (IRF). The IRF and the decays are normalized to the emission maximum and have a constant 0.2 offset for displaying purposes. The crystals were grown with 10% MPD, 10 mM spermine, 50 mM MOPS and **A)** 10 mM $LiNO_3$, **B)** 100 mM $LiNO_3$ or **C)** 200 mM $LiNO_3$. Every decay was tail-fitted with 2 exponents, and the mean values of the intensity-weighted average decay times, <τ_w>, are **A)** 3.38 ns, **B)** 3.34 ns and **C)** 2.82 ns.

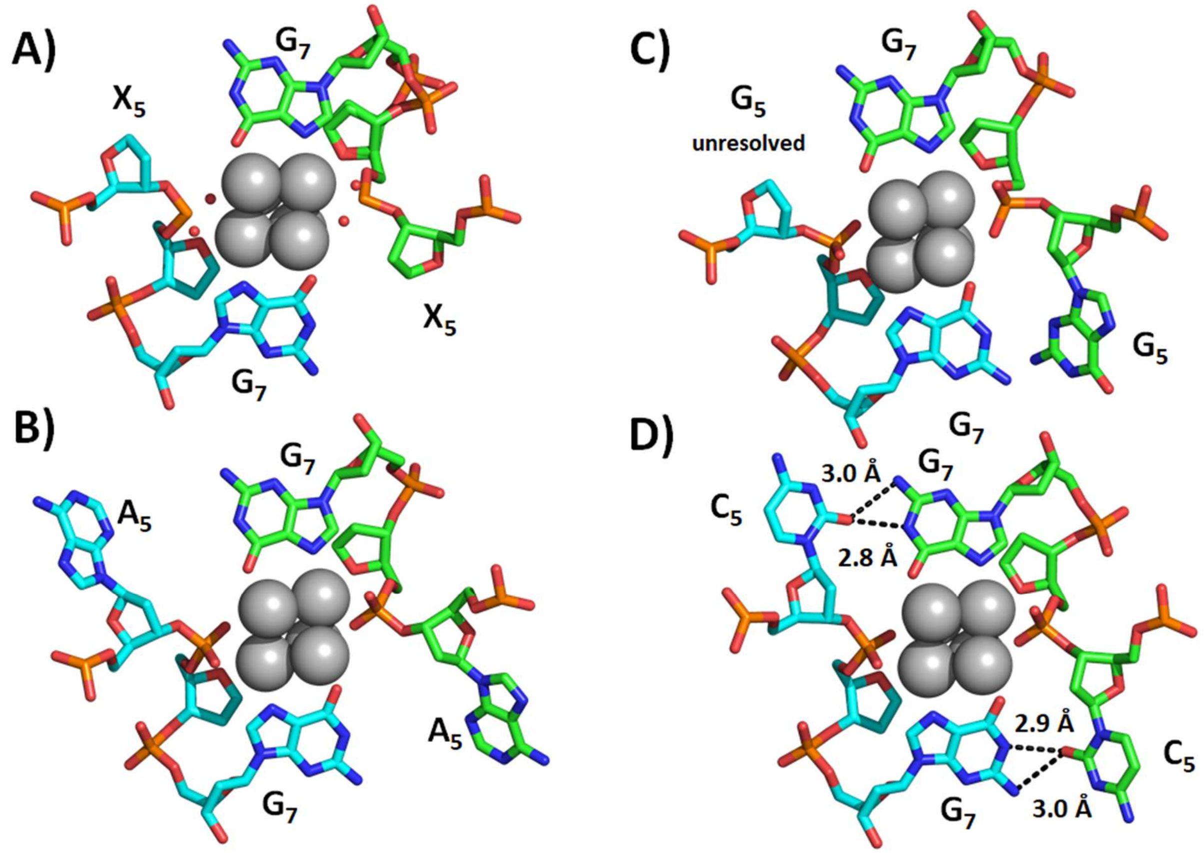

Figure S5. Comparison of positions 5 and 7 for **A)** X5, **B)** A5, **C)** G5-NIR and **D)** C5 mutations. Black dashed lines represent the hydrogen bonds between nucleobases with the related distances. The red dots in A) are oxygen atoms of the phosphate groups.

I7-I9 Crystals

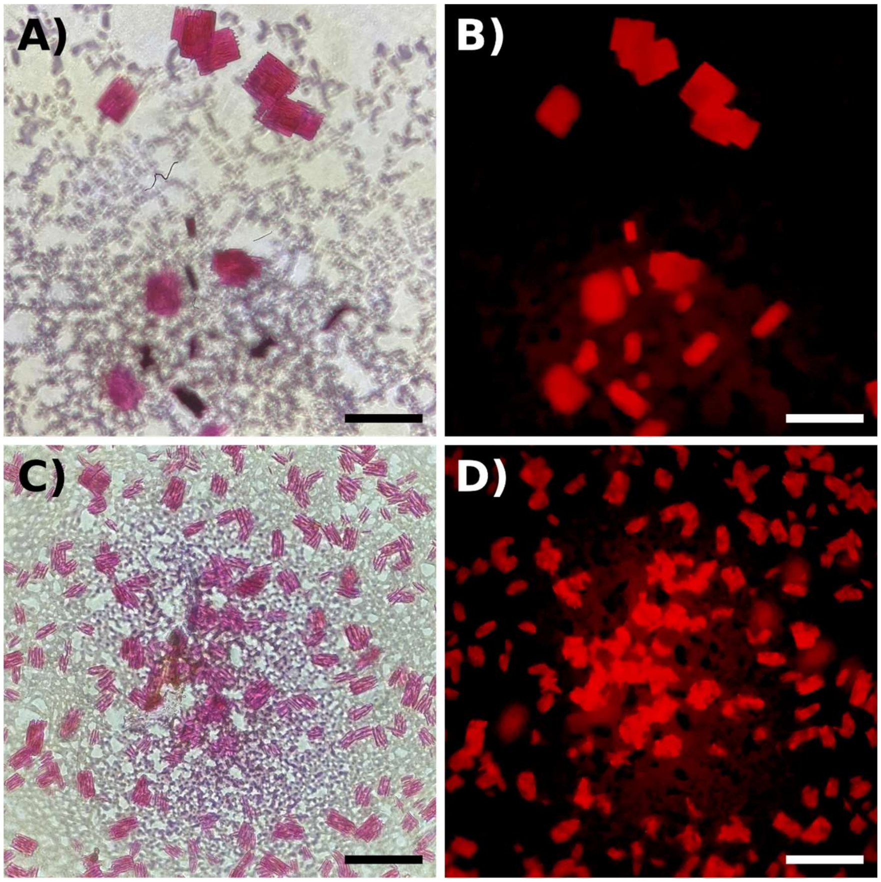

<u>Figure S6.</u> **A, C)** Bright-field and **B, D)** fluorescence images of I7-I9_DNA-Ag_{16}NC crystals at 20x magnification. To avoid saturation, the fluorescence was measured with a 3 ND filter in front of the excitation lamp. Images were taken circa 5 months after starting the crystallization. The crystals were grown with 10% PEG 3350, 10 mM spermine, 50 mM MOPS and **A, B)** 10 mM $Sr(NO_3)_2$ or **C, D)** 10 mM NH_4NO_3. Scalebar: 50 µm.

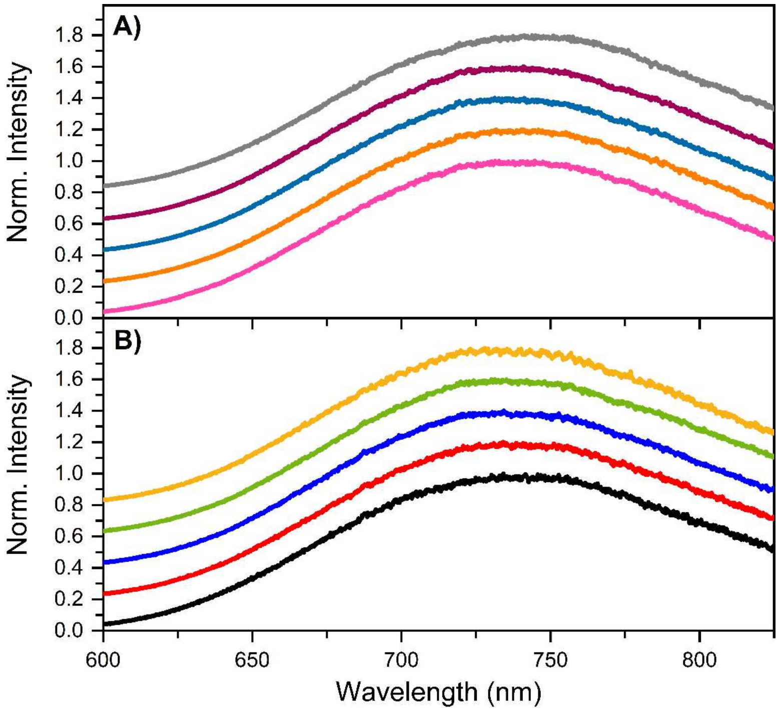

Figure S7. Emission spectra of I7-I9_DNA-Ag_{16}NC crystals, recorded with a confocal microscope exciting at 520 nm. The spectra are normalized to the emission maximum and have a constant 0.2 offset for displaying purposes. The crystals were grown with 10% PEG 3350, 10 mM spermine, 50 mM MOPS and **A)** 10 mM NH_4NO_3 or **B)** 10 mM $Sr(NO_3)_2$.

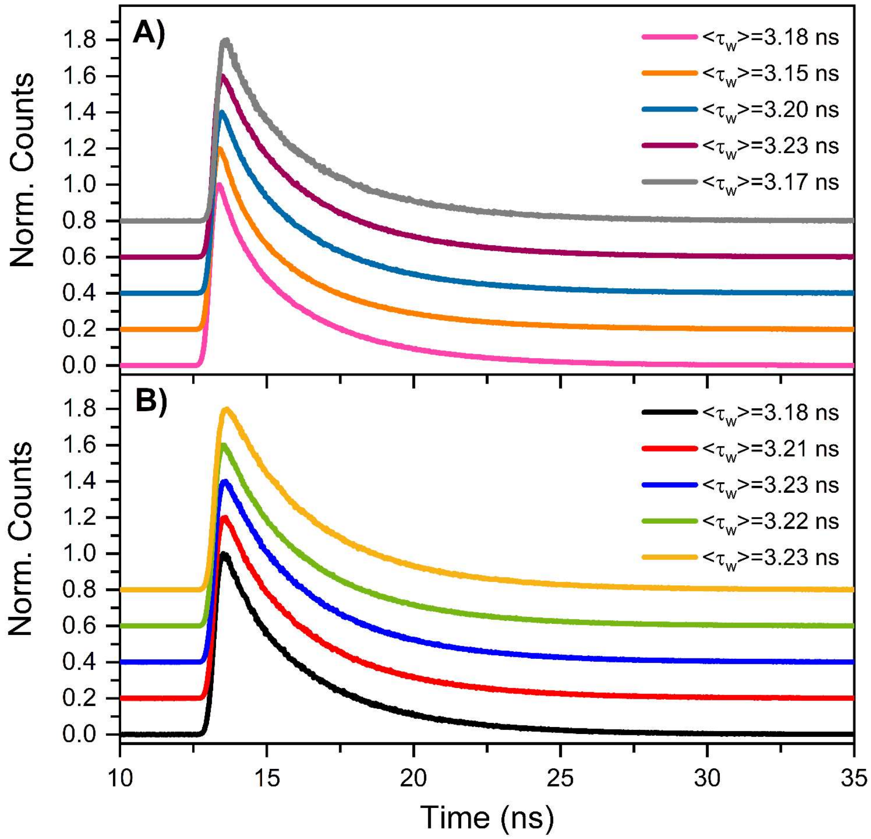

Figure S8. Fluorescence decay curves of I7-I9_DNA-Ag_{16}NC crystals, exciting at 520 nm. The decays are normalized to the emission maximum and have a constant 0.2 offset for displaying purposes. The crystals were grown with 10% PEG 3350, 10 mM spermine, 50 mM MOPS and **A)** 10 mM NH_4NO_3 or **B)** 10 mM $Sr(NO_3)_2$. Each decay was tail-fitted with a bi-exponential function, and the mean values of the intensity-weighted average decay times, $\langle\tau_w\rangle$, are **A)** 3.19 ns and **B)** 3.21 ns.

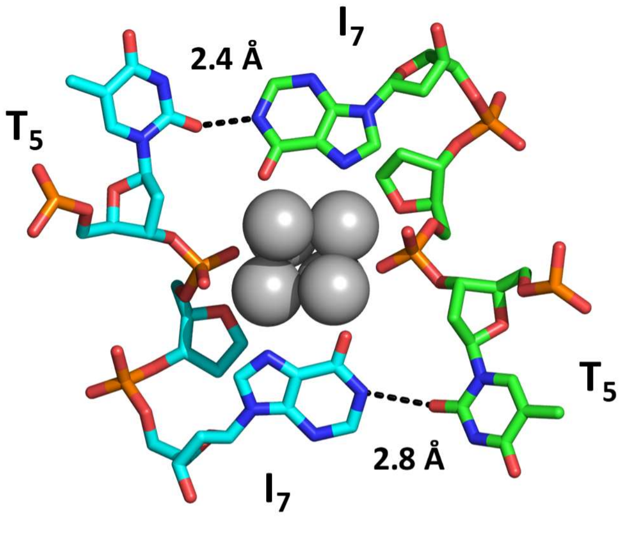

Figure S9. Cross-section of the region around positions 5 and 7 for the I7-I9 mutant. Selected hydrogen bonds between nucleobases are depicted as black dashed lines.

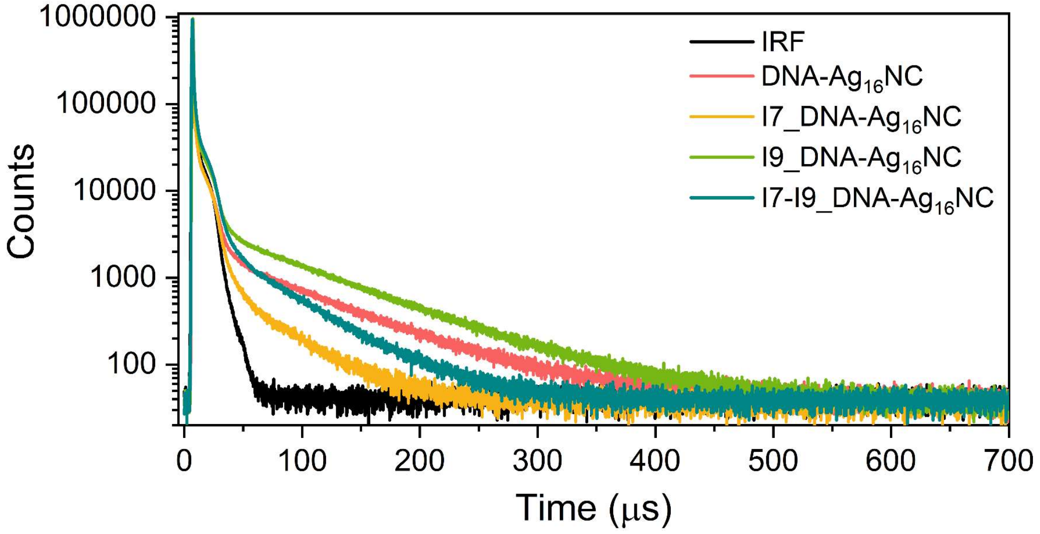

Figure S10. Microsecond decay curves of DNA-Ag_{16}NC and inosine mutants synthesized in a 10 mM NH_4OAc H_2O solution and measured in a 10 mM NH_4OAc D_2O solution at 25 ^{o}C. The decays were monitored at 820 nm, exciting at 508 nm with a Xenon flash lamp (repetition rate = 300 Hz). All decays were tail-fitted with a mono-exponential function, and the decay times ($<\tau_{\mu s}>$) are reported in Table 2. The black trace is the IRF, *i.e.,* the instrument response function.

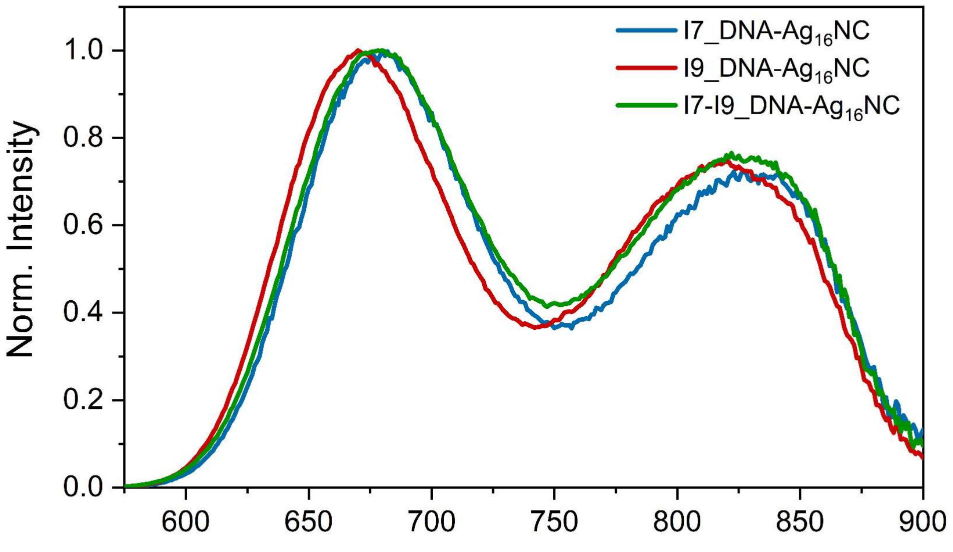

Figure S11. Normalized emission spectra of inosine mutants synthesized in a 10 mM NH_4OAc H_2O solution and measured in a 10 mM NH_4OAc D_2O solution at -196 ^{o}C. The samples were excited at 507.5 nm with a ps-pulsed laser, and two filters were used: a 510 nm band-pass filter (Semrock, FF02-510/10-25) in the excitation path and a 532 nm long-pass (Semrock, BLP01-532R-25) in the emission path.

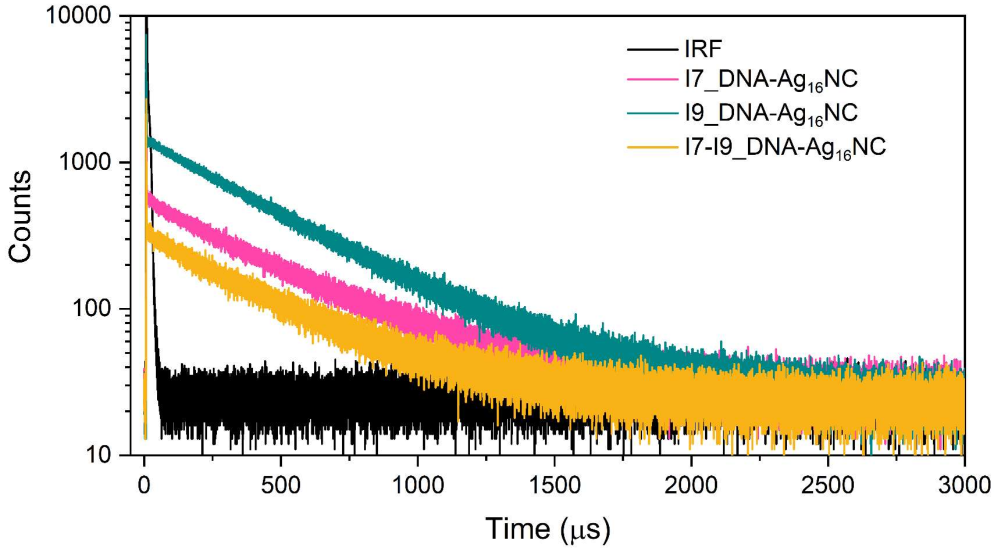

<u>Figure S12.</u> Microsecond decay curves measured at 820 nm for all inosine mutants synthesized in a 10 mM NH_4OAc H_2O solution and measured in a 10 mM NH_4OAc D_2O solution at -196 ^oC. The samples were excited at 508 nm with a Xe flash lamp (repetition rate = 300 Hz). Two filters were utilized to reduce the scattering: a 510 nm band-pass filter (Semrock, FF02-510/10-25) in the excitation path and a 532 nm long-pass (Semrock, BLP01-532R-25) in the emission path.

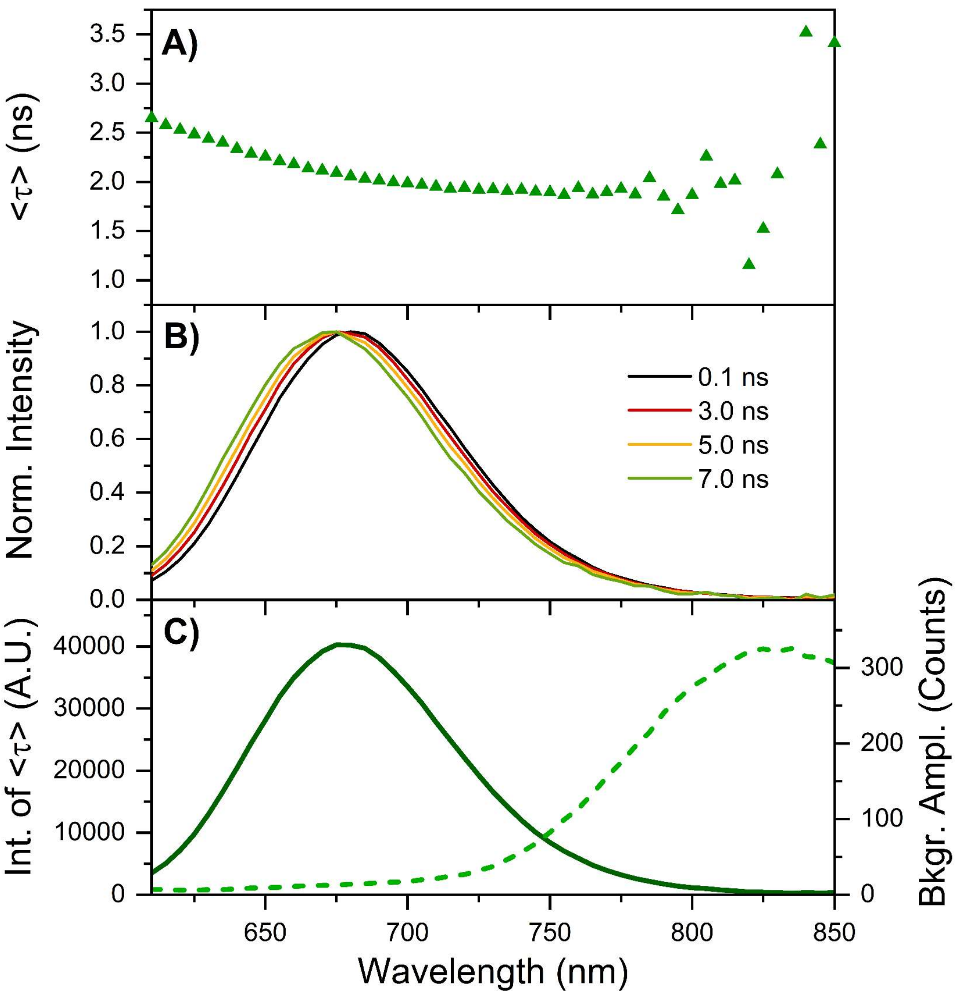

<u>Figure S13.</u> Time-resolved measurements of I7_DNA-Ag_{16}NCs synthesized in a 10 mM NH_4OAc H_2O solution and measured in a 10 mM NH_4OAc D_2O solution, carried out at -196 ^oC. The sample was excited at 507.5 nm with a ps-pulsed laser. **A)** Intensity-weighted average decay time, <τ>, as a function of emission wavelength. It is worth noticing that <τ> increases from 700 to 630 nm. **B)** Normalized time-resolved emission spectra as a function of emission wavelength. The blue-shift in the nanosecond timescale is due to the above-mentioned rise of <τ> below 700 nm. **C)** Normalized emission intensity of <τ> (solid line) and background amplitude of the decays (dashed line) as a function of emission wavelength. The background amplitude is a proxy for the µs-lived emission.

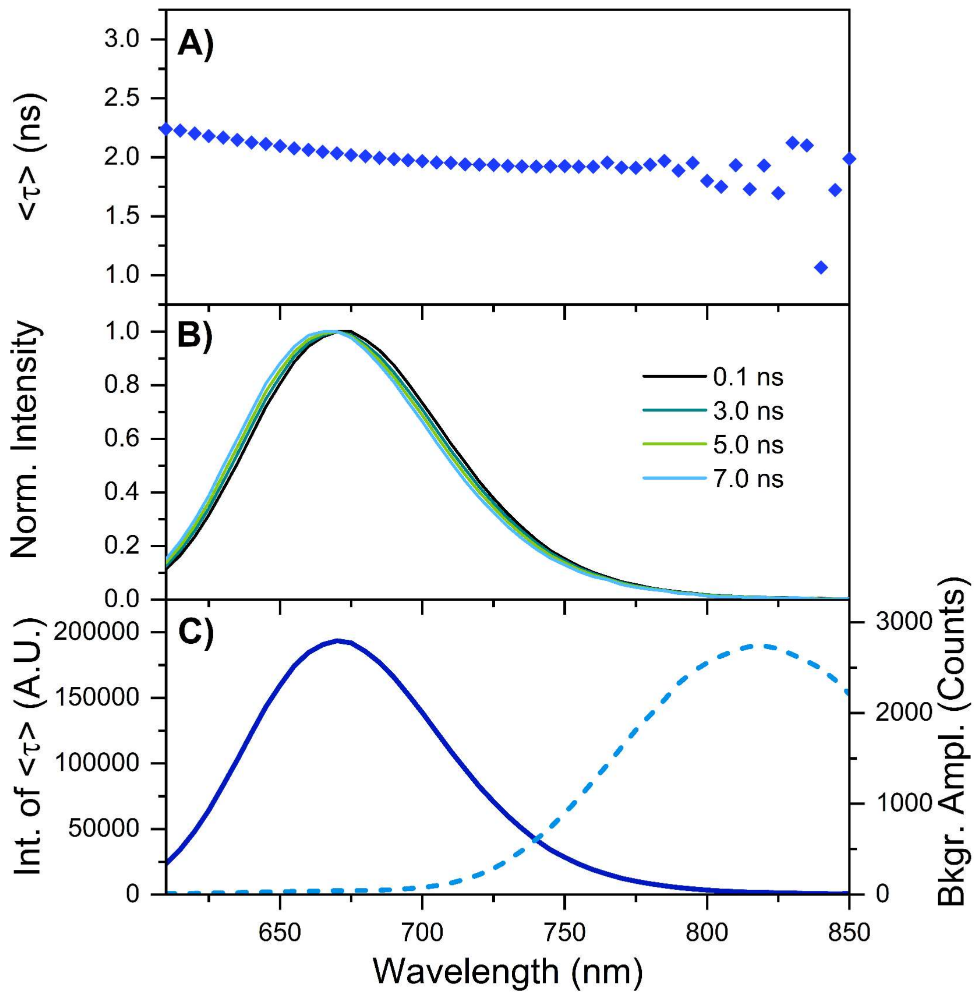

Figure S14. Time-resolved measurements of I9_DNA-Ag_{16}NCs synthesized in a 10 mM NH_4OAc H_2O solution and measured in a 10 mM NH_4OAc D_2O solution, performed at -196 ^oC. The sample was excited at 507.5 nm with a ps-pulsed laser. **A)** Intensity-weighted average decay time, <τ>, as a function of emission wavelength. It is worth noticing that <τ> increases from 680 to 630 nm. **B)** Normalized time-resolved emission spectra as a function of emission wavelength. The blue-shift in the nanosecond timescale is due to the above-mentioned rise of <τ> below 680 nm. **C)** Normalized emission intensity of <τ> (solid line) and background amplitude of the decays (dashed line) as a function of emission wavelength. The background amplitude is a proxy for the µs-lived emission.

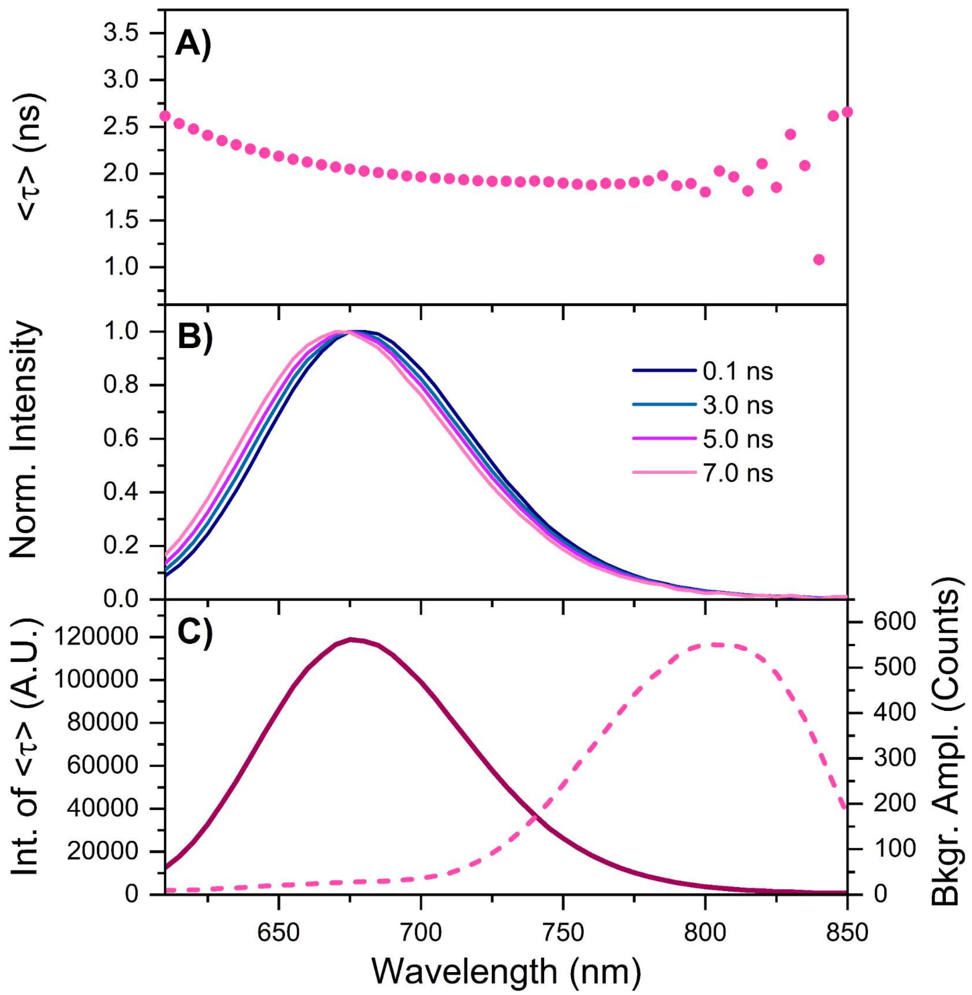

Figure S15. Time-resolved measurements of I7-I9_DNA-Ag_{16}NCs synthesized in a 10 mM NH_4OAc H_2O solution and measured in a 10 mM NH_4OAc D_2O solution, carried out at -196 ^{o}C. The sample was excited at 507.5 nm with a ps-pulsed laser. **A)** Intensity-weighted average decay time, <τ>, as a function of emission wavelength. It is worth noticing that <τ> increases from 700 to 630 nm. **B)** Normalized time-resolved emission spectra as a function of emission wavelength. The blue-shift in the nanosecond timescale is due to the above-mentioned rise of <τ> below 700 nm. **C)** Normalized emission intensity of <τ> (solid line) and background amplitude of the decays (dashed line) as a function of emission wavelength. The background amplitude is a proxy for the µs-lived emission.

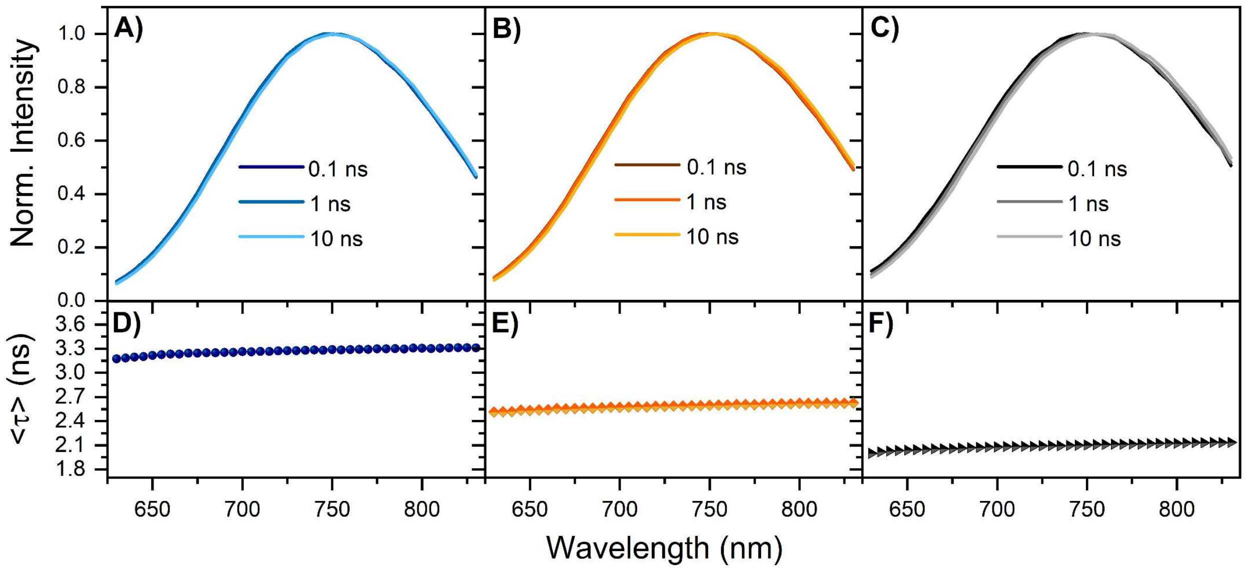

Figure S16. **A) → C)** Time-resolved emission spectra and **D) → F)** intensity-weighted average decay times, <τ>, of I7_DNA-Ag_{16}NCs in 10 mM NH_4OAc aqueous solution as a function of emission wavelength. The data was measured at three different temperatures: **A**, **D)** 5 ^{o}C, **B**, **E)** 25 ^{o}C, and **C**, **F)** 40 ^{o}C, exciting the sample at 507.5 nm with a ps-pulsed laser.

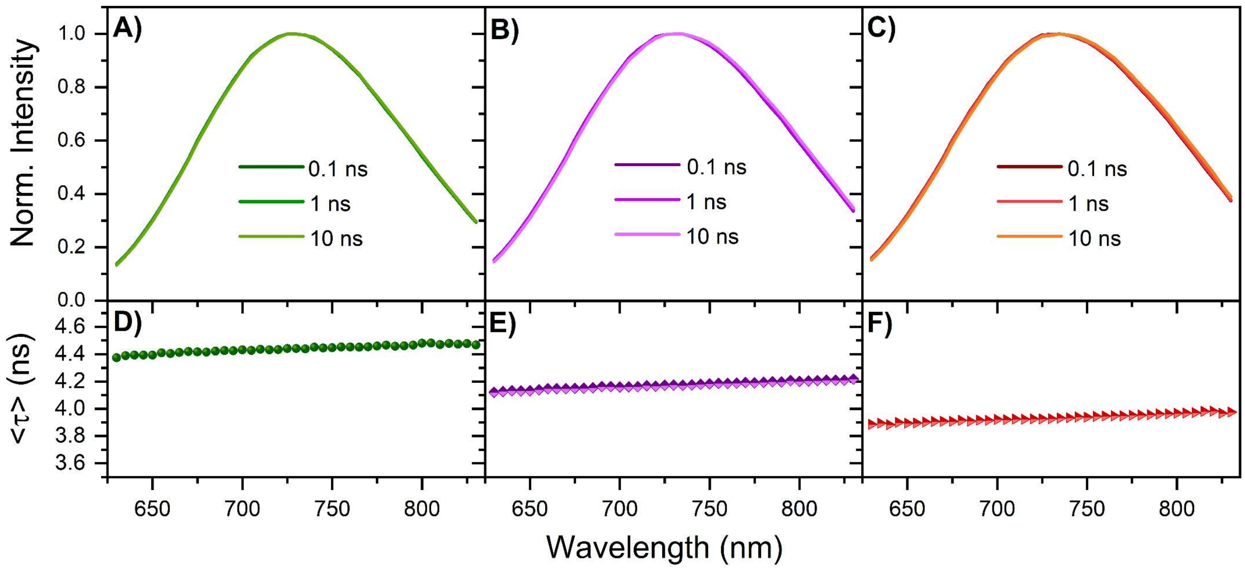

Figure S17. **A) → C)** Time-resolved emission spectra and **D) → F)** intensity-weighted average decay times, <τ>, of I9_DNA-Ag_{16}NCs in 10 mM NH_4OAc aqueous solution as a function of emission wavelength. The data was recorded at three different temperatures: **A**, **D)** 5 ^{o}C, **B**, **E)** 25 ^{o}C, and **C**, **F)** 40 ^{o}C, exciting the sample at 507.5 nm with a ps-pulsed laser.

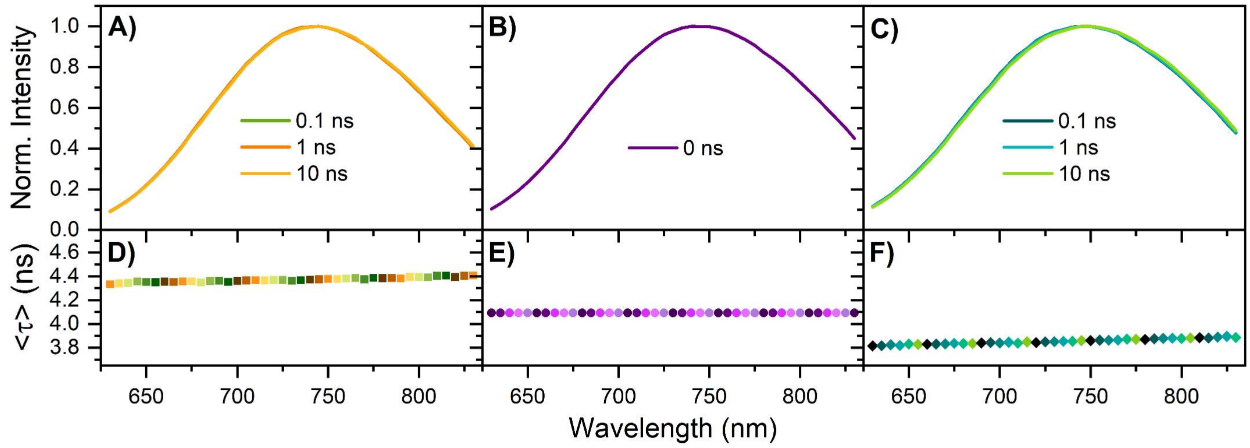

<u>Figure S18.</u> **A)** → **C)** Time-resolved emission spectra and **D)** → **F)** intensity-weighted average decay times, <τ>, of I7-I9_DNA-Ag_{16}NCs in 10 mM NH_4OAc aqueous solution as a function of emission wavelength. The data was recorded at three different temperatures: **A**, **D)** 5 ^oC, **B**, **E)** 25 ^oC, and **C**, **F)** 40 ^oC, exciting the sample at 507.5 nm with a ps-pulsed laser. The decay curves at 25 ^oC could be fitted globally with a mono-exponential model.

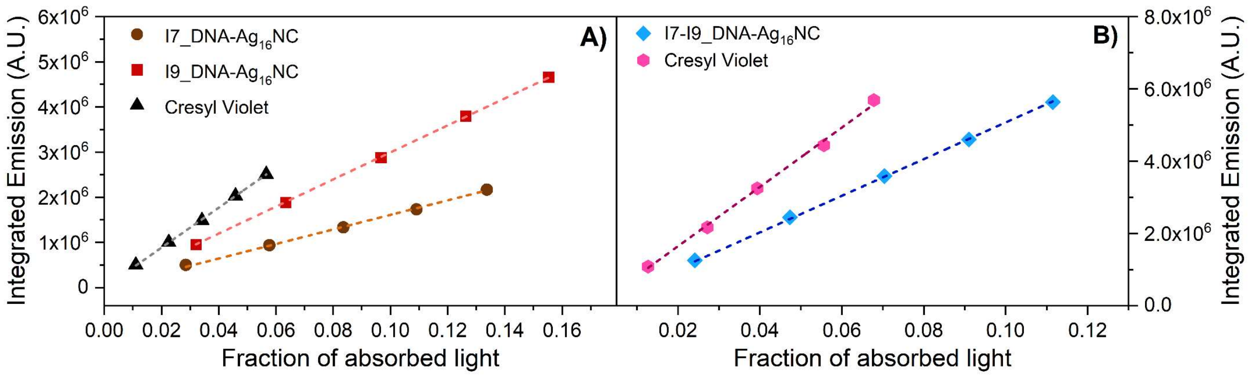

<u>Figure S19.</u> Integrated emission spectra as a function of absorbed light at λ_{exc}= 532 nm for all inosine mutants in 10 mM NH_4OAc H_2O solution, as well as Cresyl Violet used as reference dye for quantum yield calculations. The slope values for the linearly fitted data reported in **A)** are $1.61 \cdot 10^7$ (I7_DNA-Ag_{16}NC), $2.99 \cdot 10^7$ (I9_DNA-Ag_{16}NC), and $4.43 \cdot 10^7$ (Cresyl Violet). The slopes of the linear fits in **B)** are $5.07 \cdot 10^7$ (I7-I9_DNA-Ag_{16}NC) and $8.22 \cdot 10^7$ (Cresyl Violet). The measurements in **A)** and **B)** were performed on different days, hence the presence of twice the data for the reference compound and the discrepancy in the slope value.

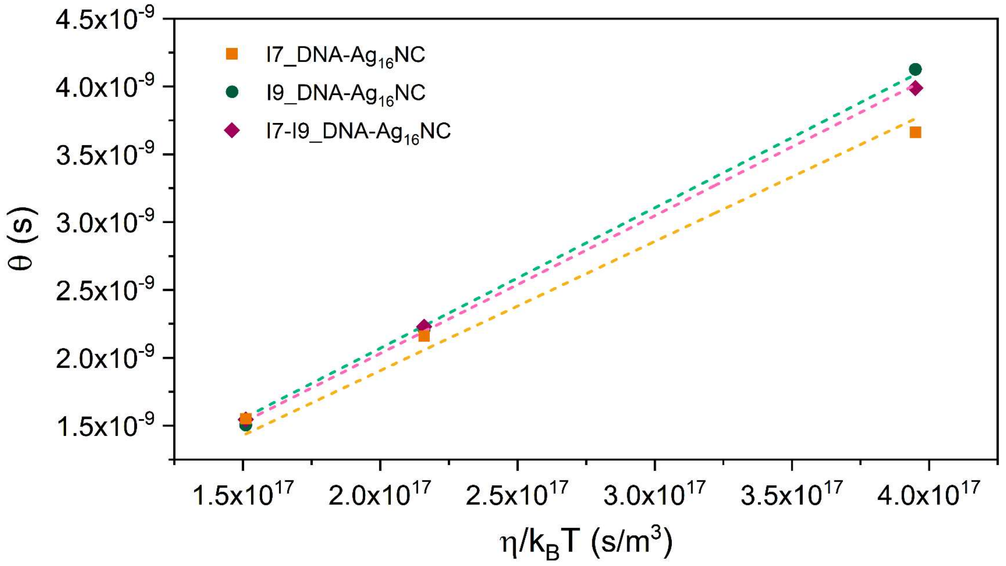

Figure S20. Rotational correlation times of all inosine mutants in 10 mM NH_4OAc H_2O solution as a function of η/k_BT (T= 5, 25, 40 °C). The resulting slopes were used to calculate the hydrodynamic volumes reported in Table 1.

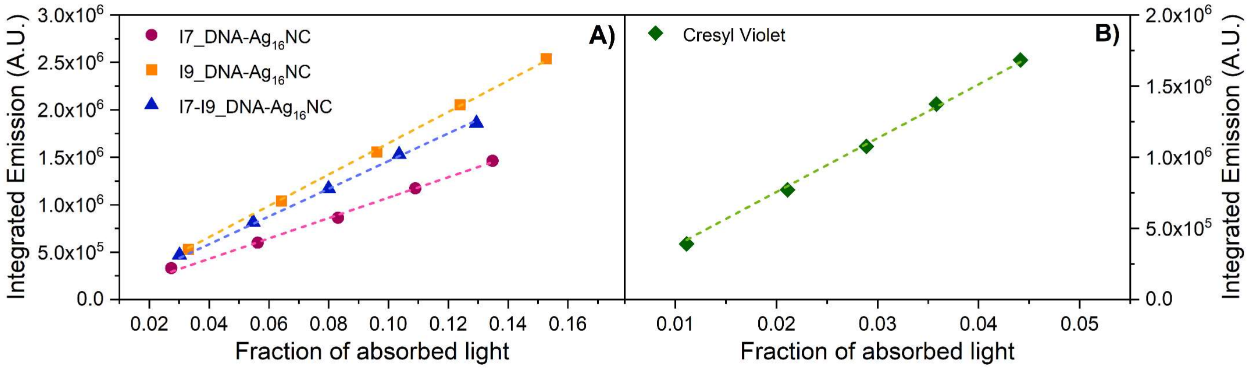

Figure S21. A) Integrated emission spectra as a function of absorbed light at λ_{exc}= 532 nm for all inosine-modified DNA-Ag_{16}NCs synthesized in a 10 mM NH_4OAc H_2O solution and measured in a 10 mM NH_4OAc D_2O solution at 25 °C. **B)** Same plot for Cresyl Violet in absolute ethanol at 25 °C. The slopes for the linearly fitted data are $1.08 \cdot 10^7$ (I7_DNA-Ag_{16}NC), $1.65 \cdot 10^7$ (I9_DNA-Ag_{16}NC), $1.46 \cdot 10^7$ (I7-I9_DNA-Ag_{16}NC) and $3.78 \cdot 10^7$ (Cresyl Violet). The measurements in **A)** and **B)** were carried out the same day.

Table S1. Crystal data and statistics of data collections and structure refinements for the I7 and I7-I9 mutants.

Crystal code (PDB code)	I7 (7XLV)	I7-I9 (7XLW)
Crystal data		
Space group	$P2_12_12_1$	$P1$
Unit cell (Å, °)	a = 24.2, b = 41.5, c = 45.3	a = 28.2, b = 49.7, c = 55.9<br>α = 74.0, β = 90.4, γ = 83.7
No. of DNA-AgNC in AU [a]	1	6
Data collection		
Beamline	BL-17A in Photon Factory	BL-17A in Photon Factory
Wavelength (Å)	0.98	0.9
Resolution (Å)	24.0 – 1.1	41.7 – 1.9
of the outer shell (Å)	1.13 – 1.1	1.94 – 1.9
Unique reflections	35080	22032
Completeness (%)	97.5	95.3
in the outer shell (%)	94.9	92.9
R_{merge} [b] (%)	-	8.3
in the outer shell (%)	-	37.7
R_{anom} [c] (%)	8.4	-
in the outer shell (%)	39.7	-
Redundancy	3.4	3.6
in the outer shell	3.1	3.6
$I/\sigma(I)$	9.9	10.8
in the outer shell	3.0	3.2
Structure refinement		
Resolution range (Å)	30.6 – 1.1	41.7 – 1.9
Used reflections	35042	21979
R-factor [d] (%)	10.2	15.0
R_{free} [e] (%)	11.0	18.3
R.m.s.d. bond length (Å)	0.010	0.008
R.m.s.d. bond angles (°)	1.4	1.4

[a] Number of molecules, atoms or ions in the asymmetric unit.

[b] $R_{merge} = 100 \times \Sigma_{hklj}|I_{hklj} - \langle I_{hklj}\rangle| / \Sigma_{hklj}\langle I_{hklj}\rangle$.

[c] $R_{anom} = 100 \times \Sigma_{hklj}|I_{hklj}(+) - I_{hklj}(-)| / \Sigma_{hklj}[I_{hklj}(+) + I_{hklj}(-)]$.

[d] R-factor = $100 \times \Sigma||F_o| - |F_c|| / \Sigma|F_o|$, where $|F_o|$ and $|F_c|$ are optimally scaled observed and calculated structure factor amplitudes, respectively.

[e] Calculated using a random set containing 10% of observations.

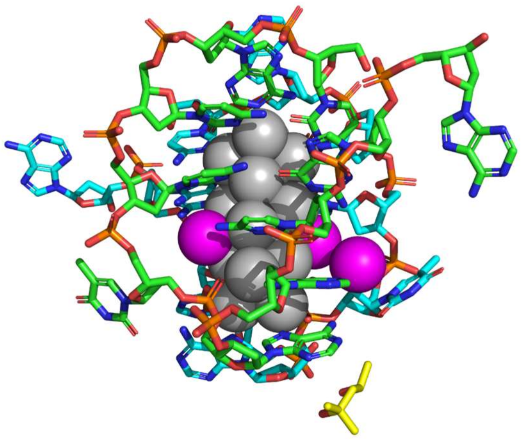

Figure S22. Unit cell of the I7 mutant. Note that 2-Methyl-2,4-pentanediol (yellow compound) co-crystallized. The magenta spheres represent silvers with an occupancy below 1.

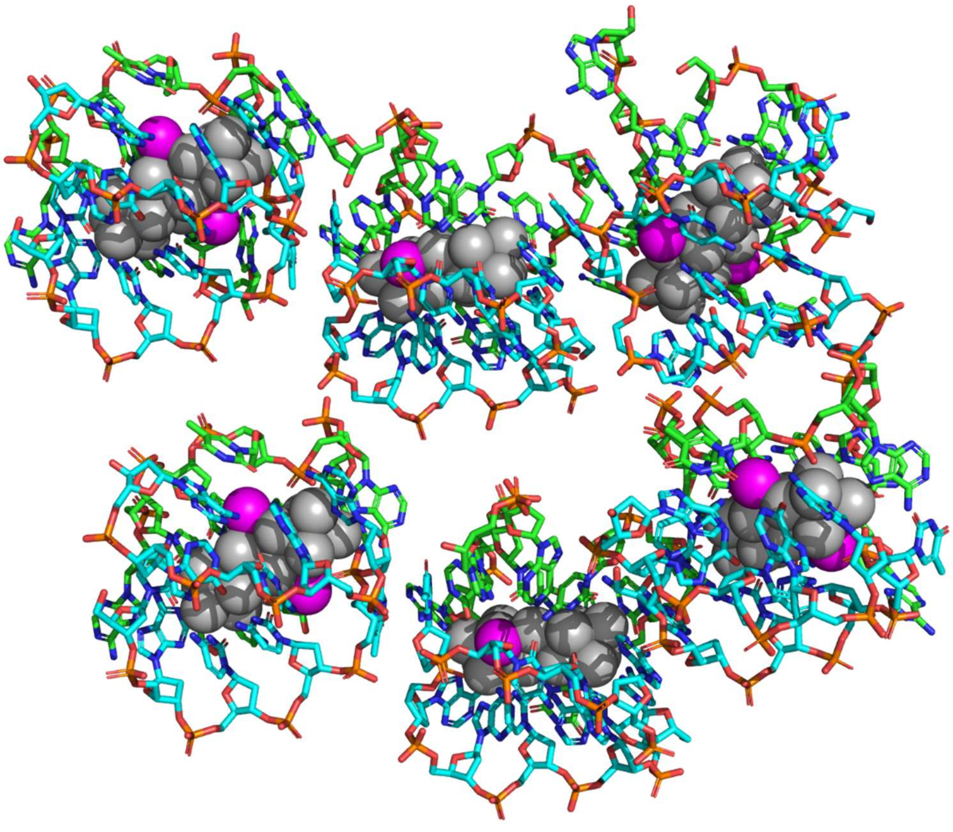

Figure S23. Unit cell of the I7-I9 mutant comprising six I7-I9_DNA-Ag_{16}NCs. Every subunit shows the same AgNC core, but slightly different bond lengths and positions of the nucleobases. The cross-sections reported in Figure 3 and Figure S9 are from one of these six DNA-AgNCs. The magenta spheres represent silvers with an occupancy below 1.

References

1. S. A. Bogh, M. R. Carro-Temboury, C. Cerretani, S. M. Swasey, S. M. Copp, E. G. Gwinn and T. Vosch, *Methods and applications in fluorescence*, 2018, **6**, 024004.
2. A. M. Brouwer, *Pure and Applied Chemistry*, 2011, **83**, 2213-2228.
3. J. R. Lakowicz, *Principles of fluorescence spectroscopy*, Springer, 2006.
4. M. Liisberg, S. Krause, C. Cerretani and T. Vosch, *Chemical Science*, 2022, **13**, 5582-5587.
5. W. Kabsch, *Acta Crystallographica Section D: Biological Crystallography*, 2010, **66**, 133-144.
6. P. D. Adams, P. V. Afonine, G. Bunkóczi, V. B. Chen, I. W. Davis, N. Echols, J. J. Headd, L.-W. Hung, G. J. Kapral and R. W. Grosse-Kunstleve, *Acta Crystallographica Section D: Biological Crystallography*, 2010, **66**, 213-221.
7. R. W. Grosse-Kunstleve and P. D. Adams, *Acta Crystallographica Section D: Biological Crystallography*, 2003, **59**, 1966-1973.
8. T. C. Terwilliger, P. D. Adams, R. J. Read, A. J. McCoy, N. W. Moriarty, R. W. Grosse-Kunstleve, P. V. Afonine, P. H. Zwart and L.-W. Hung, *Acta Crystallographica Section D: Biological Crystallography*, 2009, **65**, 582-601.
9. P. Emsley and K. Cowtan, *Acta Crystallographica Section D: Biological Crystallography*, 2004, **60**, 2126-2132.
10. P. Emsley, B. Lohkamp, W. G. Scott and K. Cowtan, *Acta Crystallographica Section D: Biological Crystallography*, 2010, **66**, 486-501.
11. P. V. Afonine, R. W. Grosse-Kunstleve, N. Echols, J. J. Headd, N. W. Moriarty, M. Mustyakimov, T. C. Terwilliger, A. Urzhumtsev, P. H. Zwart and P. D. Adams, *Acta Crystallographica Section D: Biological Crystallography*, 2012, **68**, 352-367.
12. M. B. Liisberg, Z. Shakeri Kardar, S. M. Copp, C. Cerretani and T. Vosch, *The Journal of Physical Chemistry Letters*, 2021, **12**, 1150-1154.
